# Active ingredients of traditional Chinese medicine inhibit NOD-like receptor protein 3 inflammasome: a novel strategy for preventing and treating heart failure

**DOI:** 10.3389/fimmu.2025.1520482

**Published:** 2025-01-24

**Authors:** Ruifang Lin, Yunfeng Yu, Lixin Du, Zehui Ding, Ziyan Wang, Jiaming Wei, Zhihua Guo

**Affiliations:** ^1^ College of Chinese Medicine, Hunan University of Chinese Medicine, Changsha, Hunan, China; ^2^ Hunan Key Laboratory of Colleges and Universities of Intelligent TCM Diagnosis and Preventive Treatment of Chronic Diseases, Hunan University of Chinese Medicine, Changsha, China; ^3^ First Clinical College of Chinese Medicine, Hunan University of Chinese Medicine, Changsha, Hunan, China; ^4^ College of Pharmacy, Hunan University of Chinese Medicine, Changsha, Hunan, China

**Keywords:** NLRP3, inflammasome, heart failure, active ingredient, traditional Chinese medicine

## Abstract

Heart failure (HF) has emerged as a significant global public health challenge owing to its high rates of morbidity and mortality. Activation of the NOD-like receptor protein 3 (NLRP3) inflammasome is regarded as a pivotal factor in the onset and progression of HF. Therefore, inhibiting the activation of the NLRP3 inflammasome may represent a promising therapeutic approach for preventing and treating HF. The active ingredients serve as the foundation for the therapeutic effects of traditional Chinese medicine (TCM). Recent research has revealed significant advantages of TCM active ingredients in inhibiting the activation of the NLRP3 inflammasome and enhancing cardiac structure and function in HF. The study aimed to explore the impact of NLRP3 inflammasome activation on the onset and progression of HF, and to review the current advancements in utilizing TCM active ingredients to inhibit the NLRP3 inflammasome for preventing and treating HF. This provides a novel perspective for the future development of precise intervention strategies targeting the NLRP3 inflammasome to prevent and treat HF.

## Graphical Abstract

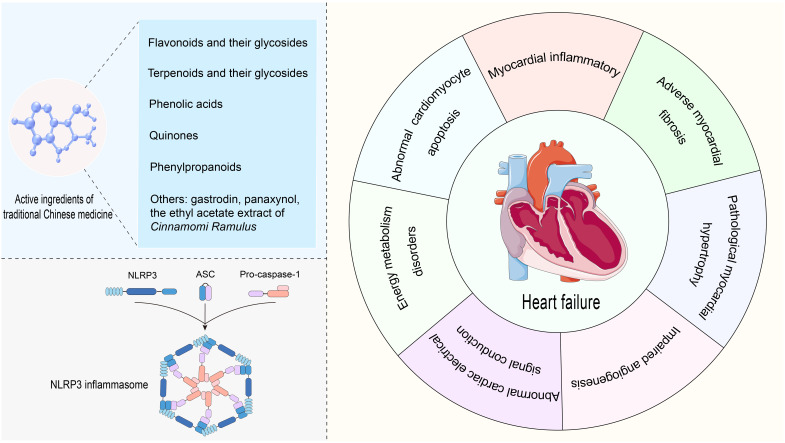

## 1 Introduction

Heart failure (HF) is the terminal stage of the progression of diverse functional or organic cardiovascular diseases, characterized by impaired ventricular filling and ejection capacity, with prevalent risk factors including hypertension, myocardial infarction, and myocardial disease (1). Epidemiological studies have indicated a global total of > 64 million patients with HF, with a prevailing trend toward a younger age of onset (2, 3). HF has emerged as a significant public health issue, posing risks to human health and escalating societal burden (2). Currently, the standard pharmacological interventions for HF management include angiotensin receptor-neprilysin inhibitors, angiotensin-converting enzyme inhibitors, angiotensin receptor blockers, sodium-glucose co-transporter 2 inhibitors, beta blockers, aldosterone receptor antagonists, and diuretics (4). Although they ameliorate HF to a certain extent, uncertainties persist concerning their long-term effects, and the potential adverse events associated with long-term medication are concerning. Therefore, there is an urgent need to develop safe and effective therapeutic strategies. HF is a clinical syndrome encompassing a range of complex pathological processes, including myocardial inflammation, myocardial fibrosis, myocardial hypertrophy, impaired angiogenesis, abnormal cardiac electrical signal conduction, energy metabolism disorders, and abnormal cardiomyocyte apoptosis (5–9). The activation of the NOD-like receptor protein 3 (NLRP3) inflammasome plays a crucial role in driving these pathological changes (5–8, 10). As an intracellular multiprotein complex, persistent or excessive activation of the NLRP3 inflammasome serves as a critical driver of both the onset and progression of HF, with the extent of its activation being strongly correlated with disease severity and patient prognosis (5, 11, 12). Therefore, inhibiting the activation of the NLRP3 inflammasome holds promise as a novel breakthrough in the prevention and treatment of HF.

Traditional Chinese medicine (TCM) has been recognized as a promising therapeutic strategy for HF, owing to its ability to effectively reverse adverse cardiac remodeling, lower rehospitalization and mortality rates, and enhance the quality of life of patients (13, 14). The active ingredients of TCM are the material basis for its therapeutic effects and constitute the focal point of research on TCM. Relevant studies have indicated that the active ingredients of TCM can mitigate the onset and progression of HF by inhibiting the NLRP3 inflammasome (15–17). Thus, this study summarizes the role of NLRP3 inflammasome activation in the onset and progression of HF, as well as the current research on the use of TCM active ingredients to prevent and treat HF through targeted inhibition of the NLRP3 inflammasome, aiming to provide insights for future basic research and novel drug development.

## 2 NLRP3 inflammasome

### 2.1 Structure of the NLRP3 inflammasome

**Figure 1:**
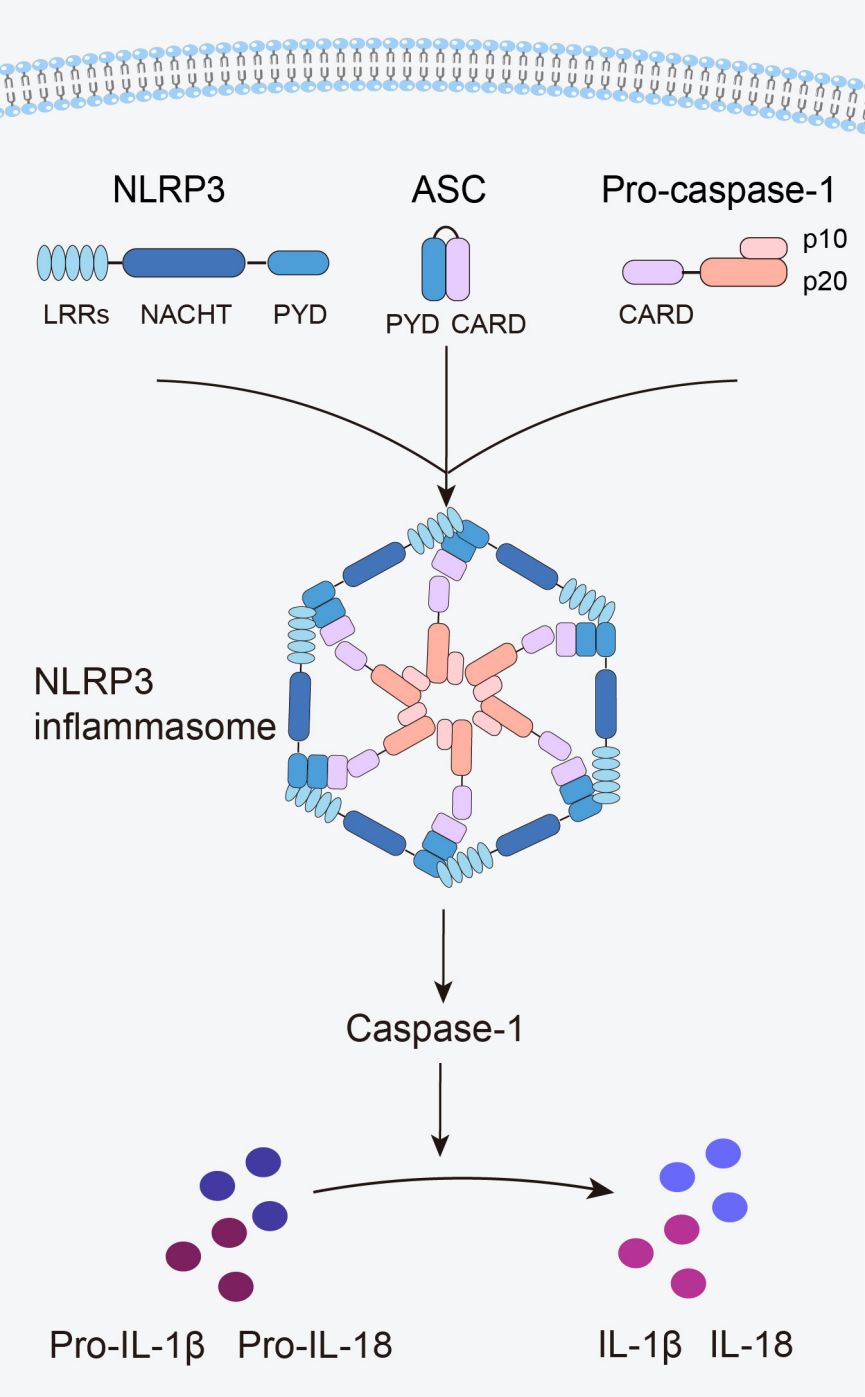
Structure of the NLRP3 inflammasome.

The innate immune system is the first line of defense in the human body. Innate immune cells activate inflammasome by recognizing pathogen-associated molecular patterns and damage-associated molecular patterns via pattern recognition receptors, subsequently initiating inflammatory responses (12). The NLRP3 inflammasome is the most widely and intensively studied inflammasome and is a multiprotein complex comprising the NLRP3 protein, apoptosis speck-like protein containing a caspase recruitment domain (ASC), and caspase-1 precursor (pro-caspase-1) (12) (**Figure 1**). NLRP3 acts as a sensor and consists of a central NACHT domain, a leucine-rich repeat (LRR) domain at the carboxyl-terminal (C-terminal), and a pyrin domain (PYD) at the amino-terminal (N-terminal). The NACHT domain primarily facilitates NLRP3 protein oligomerization and contains an adenosine triphosphatase active site, enabling the regulation of NLRP3 protein activity through adenosine triphosphate (ATP) hydrolysis (18). The LRR domain mediates protein–protein interactions and plays a crucial role in NLRP3 inflammatory signaling by recognizing and interacting with both exogenous and endogenous molecules (19). PYD recruits downstream effector signaling molecules that trigger inflammasome assembly (20). ASC functions as an adaptor with two domains: the N-terminal PYD and the C-terminal caspase activation and recruitment domain (CARD) (21). The PYD of ASC corresponds to the homotypic PYD of NLRP3 proteins, which mediates the interaction between ASC and NLRP3 proteins (20, 21). The CARD of ASC is responsible for binding pro-caspase-1 (21). Pro-caspase-1 functions as an effector and comprises three domains: the N-terminal CARD, central large catalytic subunit domain p20, and C-terminal small catalytic subunit domain p10 (22). The CARD of pro-caspase-1 is responsible for interactions with the CARD of ASC (22). Subsequently, p20 and p10 facilitate the cleavage of the interleukin-1β precursor (pro-IL-1β) and interleukin-18 precursor (pro-IL-18) into mature forms of IL-1β and IL-18.

When an organism encounters an external stimulus, NLRP3 interacts with the PYD of ASC via its PYD. The CARD of ASC recruits and binds to the CARD of pro-caspase-1, triggering self-cleavage of pro-caspase-1 to yield active caspase-1. Caspase-1 cleaves pro-IL-1β and pro-IL-18 to generate mature IL-1β and IL-18, thus initiating an inflammatory response (**Figure 1**).

### 2.2 Mechanism of NLRP3 inflammasome activation

**Figure 2:**
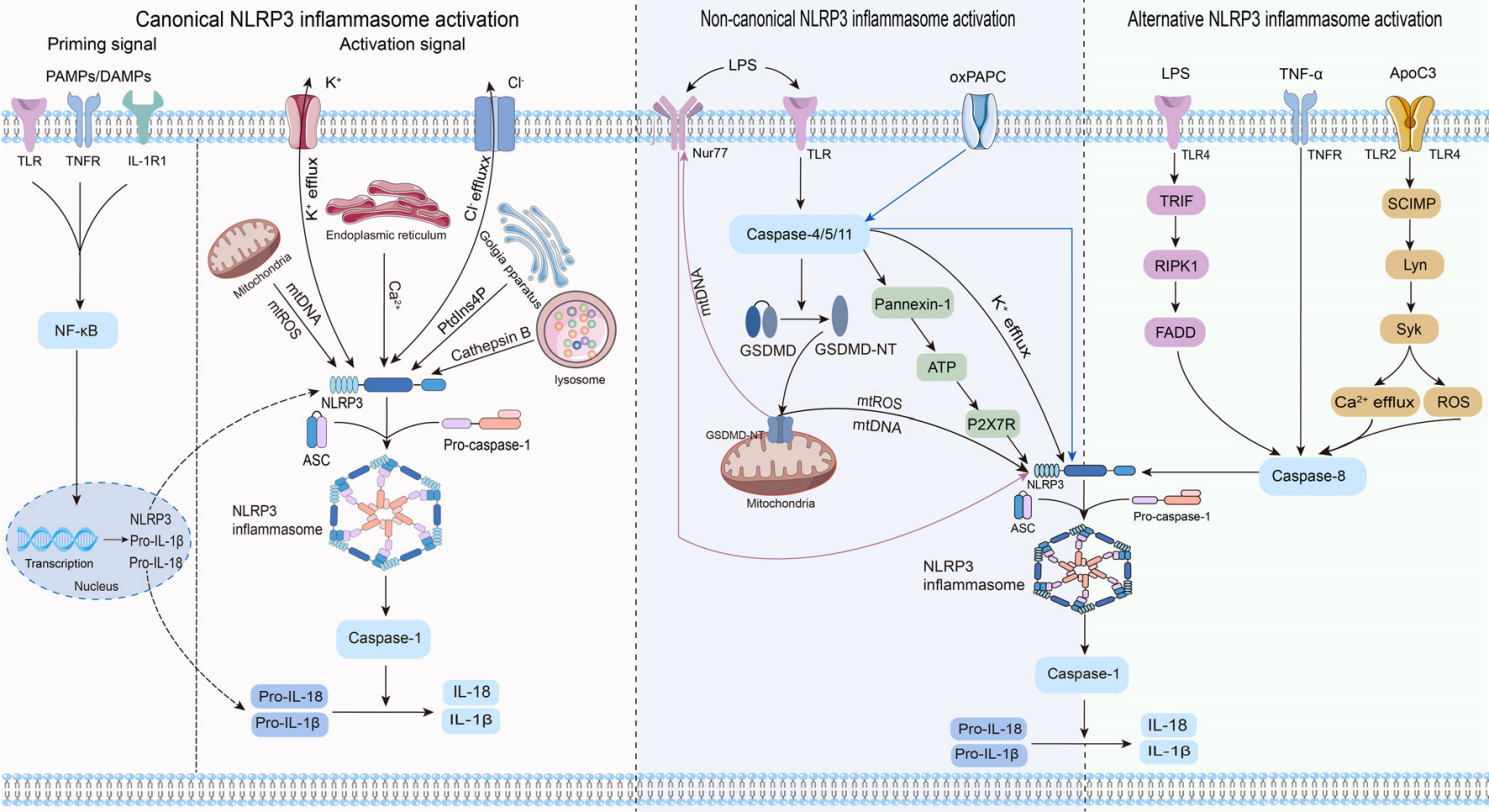
Three pathways and related mechanisms of NLRP3 inflammasome activation.

Three distinct pathways exist for NLRP3 inflammasome activation: canonical NLRP3, non-canonical NLRP3, and alternative NLRP3 inflammasome activation (23) (**Figure 2**).

#### 2.2.1 Canonical NLRP3 inflammasome activation

Canonical activation of the NLRP3 inflammasome involves two distinct processes: priming and activation (24). The priming phase involves transcriptional regulation and posttranslational modifications of NLRP3. Recognition of pathogen-associated molecular patterns or damage-associated molecular patterns by the corresponding pattern recognition receptors (25–27) triggers nuclear factor-κB (NF-κB) translocation and transcription, leading to increased expression of NLRP3, pro-IL-1β, and pro-IL-18 within the nucleus. Furthermore, priming signals trigger posttranslational modifications of NLRP3, including phosphorylation (28), ubiquitination (29), alkylation (30), S-nitrosylation (31), acetylation (32), and sumoylation (33), all of which are crucial for modulating the activation or inhibition of NLRP3. During the activation phase, NLRP3 responds to activating stimuli, subsequently initiating the assembly of the NLRP3 inflammasome, activation of caspase-1, and processing pro-IL-1β and pro-IL-18. This process ultimately produces proinflammatory cytokines IL-1β and IL-18, which are subsequently secreted into the extracellular space to trigger an inflammatory response.

Previous studies agree that the stimulus signals for NLRP3 inflammasome activation include potassium ion (K^+^) efflux (34), chloride ion (Cl^-^) efflux (35), mitochondrial dysfunction (36, 37), endoplasmic reticulum stress (38), trans-Golgi network catabolism (39), and the release of tissue protease B from damaged lysosomes (40). Remarkably, the interplay between some of these stimuli complicates the activation phase of NLRP3 (41).

#### 2.2.2 Non-canonical NLRP3 inflammasome activation

Non-canonical activation of the NLRP3 inflammasome primarily relies on the mediation of human caspase-4/5 or mouse caspase-11. CARDs of caspase-4/5/11 directly recognize lipopolysaccharides (LPS) from gram-negative bacteria, prompting their oligomerization of caspase-4/5/11 (42–44). This leads to the cleavage of GSDMD into its active form, GSDMD-NT, which in turn induces pyroptosis by creating pores in the cytoplasmic membrane (42–44). Notably, while inducing pyroptosis, caspase-4/5/11 do not directly cleave pro-IL-1β (45, 46). They are required to indirectly promote the cleavage of pro-IL-1β and the release of IL-1β through NLRP3-dependent activation of caspase-1 (45, 46). Research has revealed that caspase-4/5/11 can trigger the release of mitochondrial reactive oxygen species (mtROS) and mitochondrial DNA (mtDNA) by enhancing the pore-forming capability of GSDMD in the mitochondria, thereby contributing to the activation of the NLRP3 inflammasome (47, 48). The orphan receptor Nur77 is activated upon binding to LPS and mtDNA (48). Subsequently, Nur77 interacts with NLRP3, triggering activation of the NLRP3 inflammasome (48). After LPS stimulation, caspase-4/5/11 trigger intracellular K^+^ efflux, one of the pathways by which caspase-4/5/11 mediates NLRP3 inflammasome activation (46, 49). Furthermore, the activated caspase-11 triggers the cleavage of pannexin-1 channels, leading to the release of ATP into the extracellular environment (50). Subsequently, the P2X7 receptor (P2X7R) responds to extracellular ATP, triggering the assembly of NLRP3 inflammasome and the release of IL-1β (50). The scaffold structural domain of pro-caspase-11 facilitates the activation of NLRP3 through interaction with the LRRs and PYD of NLRP3 (51). Intriguingly, this activation is mediated by the co-induction of live gram-negative bacterial mRNA and LPS (51). In addition to LPS, oxidized phospholipids (oxPAPC) serve as endogenous ligands for caspase-11 (52). It induces the oligomerization of caspase-11 by binding to its catalytic domain in dendritic cells, thereby promoting the assembly of the NLRP3 inflammasome and inflammation (52). Nevertheless, indications suggest that oxPAPC exerts an anti-inflammatory effect as it can diminish the non-canonical activation of the macrophage NLRP3 inflammasome and dampen the inflammatory response through competitive binding of LPS to caspase-4 and caspase-11 (53). Consequently, further investigations are warranted to explore the potential divergent effects of oxPAPCs on non-canonical NLRP3 inflammasome activation across different cell types.

#### 2.2.3 Alternative NLRP3 inflammasome activation

In contrast to the previously mentioned activation pathways, the alternative activation pathway requires only one step to activate the NLRP3 inflammasome and lacks the features of canonical and non-canonical NLRP3 inflammasome activation, such as K^+^ efflux, pyroptosis, or pyroptosome formation (54). This activation pathway exhibits species specificity and has been identified exclusively in human and porcine monocytes (54). Research revealed that toll-like receptor (TLR) 4 in human monocytes triggered the NLRP3 inflammasome through the TRIF/RIPK1/FADD/caspase-8 signaling pathway upon stimulation by LPS, eliminating the need for a secondary signal for mediation (54). Tumor necrosis factor-α (TNF-α), a closely associated cytokine in psoriasis, selectively induces the initiation of the NLRP3 inflammasome through the TNFR/caspase-8 pathway even without an initial signal (55). Apolipoprotein C3 (ApoC3), an endogenous mediator, selectively triggers activation of the NLRP3 inflammasome in human monocytes (56). This process involves the formation of heterodimers between TLR2 and TLR4, initiating a pathway dependent on SCIMP/Lyn/Syk for calcium influx and ROS production, leading to caspase-8 activation and ultimately triggering activation of the NLRP3 inflammasome (56).

## 3 Role of NLRP3 inflammasome activation in the onset and progression of HF

**Figure 3:**
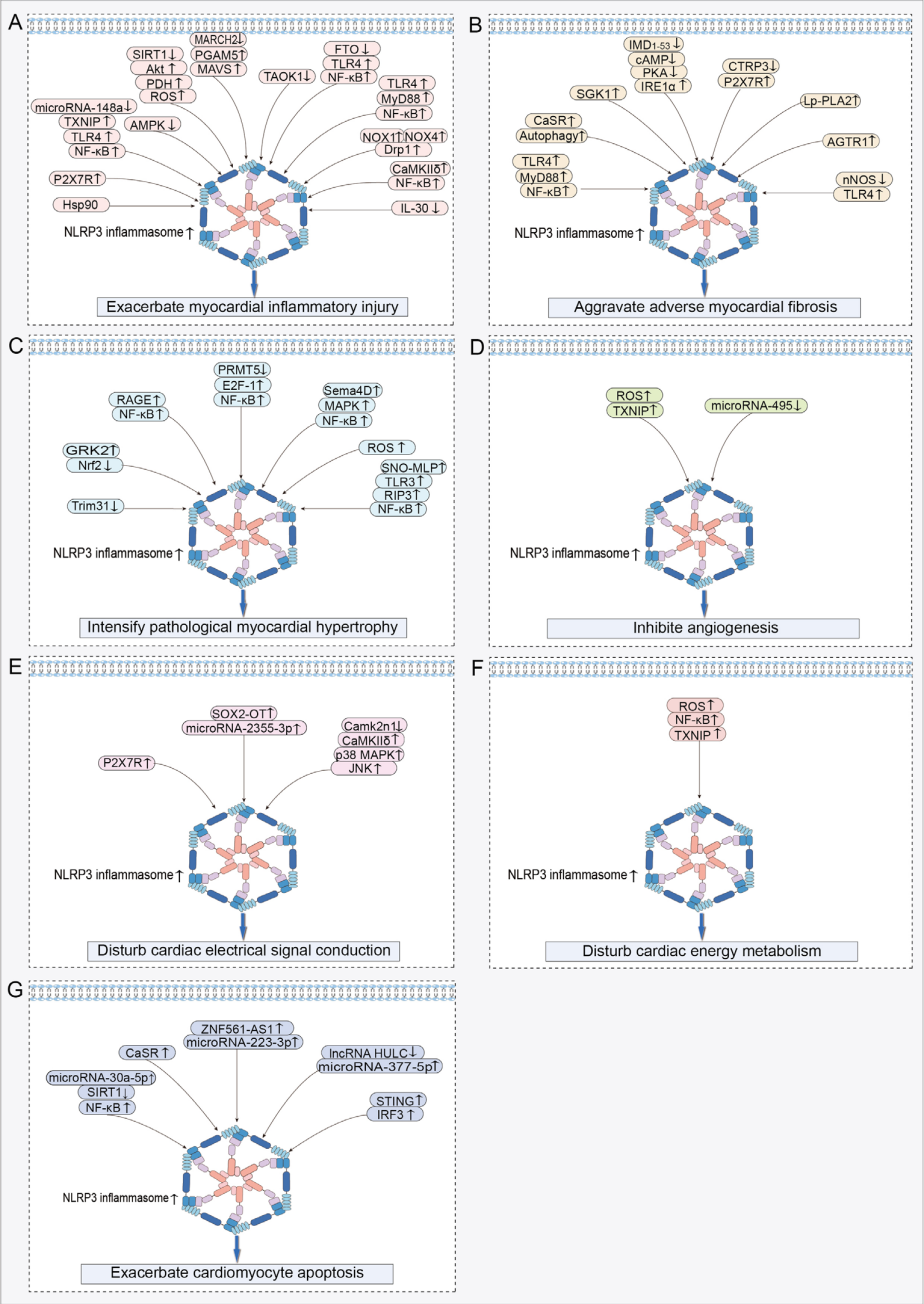
Signaling pathways regulating NLRP3 inflammasome activation in HF: **(A)** Signaling pathway exacerbating myocardial inflammatory injury. **(B)** Signaling pathway aggravating adverse myocardial fibrosis. **(C)** Signaling pathway intensifying pathological myocardial hypertrophy. **(D)** Signaling pathway inhibiting angiogenesis. **(E)** Signaling pathway disturbing cardiac electrical signal conduction. **(F)** Signaling pathway disturbing cardiac energy metabolism. **(G)** Signaling pathway exacerbating cardiomyocyte apoptosis. ↑ indicates activation; ↓ indicates inhibition.

Upon systematically reviewing studies on NLRP3 inflammasome activation in HF, we discovered that its activation promotes the onset and progression of HF by exacerbating multiple crucial pathophysiological processes. These pathological changes include myocardial inflammatory injury, adverse myocardial fibrosis, pathological myocardial hypertrophy, inhibited angiogenesis, abnormal cardiac electrical signal conduction, disturbed cardiac energy metabolism, and abnormal cardiomyocyte apoptosis (**Table 1**). Among these processes, myocardial inflammatory injury stands as a central nexus, where chronic inflammation not only directly harms the myocardium but also has the potential to initiate a cascade of events that worsen other pathological alterations in HF. Notably, the activation of the NLRP3 inflammasome involves the modulation of multiple signaling pathways, which are pivotal in mediating the aforementioned pathological processes (**Figure 3**).

**Table 1:** Role of NLRP3 inflammasome activation in the onset and progression of HF.

Effects	Targets or related signal pathways	Models		References
		*In vivo*	*In vitro*	
Exacerbate myocardial inflammatory injury	mTOR↑, NLRP3↑	Male SD rats Lipid emulsion and LADCA ligation-induced HF model	a. CD4^+^ T cells PMA/Ionomycin Mixture and IL-2-induced cell inflammation model b. THP-1 macrophages LPS and ATP-induced cell inflammation model	(59)
	Hsp90, SGT1↑, Drp1↑, NLRP3↑	Male Wistar rats LCA ligation-induced HF model	NRVMs LPS and nigericin/ATP-induced cell inflammation model	(60)
	P2X7R/NLRP3↑	Male CD1 mice LCA ligation-induced AMI model	HL-1 cells LPS and nigericin/ATP-induced cell inflammation model	(61)
	TLR4/MyD88/NF-κB/NLRP3↑	Male SD rats LADCA ligation-induced MI model	-	(62)
	NLRP3↑	Male CD-1 mice LCA ligation and release-induced MI/R model	-	(63)
	MicroRNA-148a↓, TXNIP/TLR4/NF-κB/NLRP3↑	SD rats LADCA ligation and release-induced MI/R model	NRCMs H/R-induced cell damage model	(64)
	AMPK↓, NLRP3↑	Male SD rats Langendorff perfusion-induced MI/R model	NRVMs H/R-induced cell damage model	(65)
	SIRT1↓, Akt/PDH/ROS/NLRP3↑	C57BL/6J WT, SIRT1-KO and PDH E1α-KO mice LADCA ligation and release-induced MI/R model	-	(66)
	MARCH2↓, PGAM5/MAVS/NLRP3 ↑	Male C57BL/6J WT and MARCH2-KO mice LADCA ligation and release-induced MI/R model	HL-1cells and NMCMs H/R-induced cell damage model	(67)
	TAOK1↓, YAP↓, TEAD↓, NLRP3↑	SD rats DOX intraperitoneal injection-induced HF model	H9c2 cells IL-17-induced cell inflammation model	(69)
	NLRP3↑	Male C57BL/6J mice DOX intraperitoneal injection-induced HF model	H9c2 cells DOX-induced cell toxicity model	(68)
	FTO↓, TLR4/NF-κB/NLRP3↑	Serum samples from healthy volunteers and HF patients	H9c2 cells DOX-induced cell toxicity model	(71)
	TLR4/MyD88/NF-κB/NLRP3↑	Blood samples from healthy volunteers, atrial fibrillation patients and HF patients	-	(70)
	NOX1↑, NOX4↑, Drp1↑, NLRP3↑	Female and male C57BL/6J WT, NLRP3-KO, and caspase-1-KO mice DOX intraperitoneal injection-induced DCM model	H9c2 cells and NRVCs DOX-induced cell damage model	(72)
	NLRP3↑	a. Male C57Bl/6 mice TAC-induced HF model b. Male Dahl salt-sensitive rats High-salt diet-induced HF model	Human cardiomyocytes and murine macrophages LPS-induced cell inflammation model	(74)
	CaMKIIδ/NLRP3↑	Female and male CaMKIIδ floxed and CaMKIIδ-KO mice TAC-induced cardiac pressure overload model	-	(75)
	CaMKIIδ/NF-κB/NLRP3↑	Male CaMKIIδ floxed, CaMKIIδ -KO and NLRP3-KO mice Ang II intraperitoneal injection-induced hypertension model	AMVMs and NRVMs Ang II-induced cell hypertension model	(76)
	NLRP3↑	a. Male SD rats Monocrotaline subcutaneous injection induced-PAH models b. Male SD rats Sugen-5416 ubcutaneous injection combined with H/R induced-PAH models c. Male SD rats Pulmonary artery banding induced-HF models d. RV tissue from healthy volunteers and HF patients	NRCMs and peripheral blood mononuclear cells (from rats treated with Monocrotaline) co-cultivation	(78)
	NLRP3↑	SD rats a. Monocrotaline intraperitoneal injection induced-PAH model b. LPS intraperitoneal injection induced-acute right ventricular failure model	H9c2 cells and BMDMs LPS-induced cell inflammation model	(79)
	IL-30↓, NLRP3↑	Male C57BL/6 WT and IL-30-KO mice Cecum ligation and puncture induced-myocardial dysfunction model	BMDMs LPS-induced cell inflammation model	(77)
Aggravate adverse myocardial fibrosis	NLRP3↑	Male C57BL/6 mice LCA ligation-induced myocardial infarction model	CFs Hypoxia-induced cell damage model	(86)
	TLR4/MyD88/NF-κB/NLRP3↑	Male C57BL/6J mice LADCA ligation-induced MI model	-	(87)
	CaSR↑, Beclin-1↑, LC3-II/I↑, NLRP3↑	Male Wistar rats LADCA ligation-induced MI model	Peritoneal macrophages (from MI model rats)	(88)
	NF-κB↑, NLRP3↑	Male Dahl salt-sensitive rats High-salt diet-induced HF model	-	(89)
	NF-κB/NLRP3 ↑	Male C57BL/6J mice Aortic banding-induced cardiac pressure overload model	-	(90)
	SGK1/NLRP3↑	Male B6/129S mice Ang II subcutaneous permeabilization-induced hypertension model	BMDMs and MCFs LPS and Ang II-induced cell inflammation model	(85)
	IMD_1-53_/cAMP/PKA↓, IRE1α/NLRP3↑	Male SD rats Ang II subcutaneous injection-induced myocardial fibrosis model	NRCFs Ang II-simulated fibrosis model	(91)
	CTRP3↓, P2X7R/NLRP3↑	Male WKY rats and SHRs Hypertension model	NRCFs Ang II-simulated fibrosis model	(92)
	Lp-PLA2/NLRP3↑	Male C57BL/6J mice Ang II subcutaneous permeabilization-induced hypertension model	BMDMs and RCFs LPS and Ang II-induced cell inflammation model	(84)
	NLRP3↑	Female C57BL6/J mice High-fat diet and Ang II-induced HF model	-	(93)
	NLRP3/TGF-β/Smad4↑	Male C57BL/6J mice TAC-induced pathological cardiac remodeling	-	(94)
	AGTR1/NLRP3/TGF-β1↑, AQP1↑	Male SD rats LADCA ligation-induced HF model	-	(95)
	nNOS↓, TLR4/NLRP3↑, TGF-β1/IL-1β↑	Male 129sv mice ISO subcutaneous injection-induced left ventricular fibrosis model	HCFs LPS and ATP-induced cell inflammation model	(96)
	NLRP3/ROS/TGF-β/R-Smad↑	Male C57BL6 WT, NLRP3-KO, ASC-KO, and caspase-1-KO mice Ang II subcutaneous permeabilization -induced cardiac fibrosis model	a. CFs AngII or TGF-β-simulated fibrosis model b. Peritoneal macrophages LPS and ATP-induced cell inflammation model	(97)
Intensify pathological myocardial hypertrophy	Trim31↓, NLRP3↑	Male and female C57BL/6N WT, Trim31 floxed and Trim31-KO mice ISO subcutaneous injection-induced HF model	-	(99)
	GRK2↑, Nrf2↓, NLRP3↑, OS↑	-	H9c2 cells ISO-induced cell hypertrophy model	(102)
	RAGE/NF-κB/NLRP3↑	-	H9c2 cells Ang II-induced cell hypertrophy model	(103)
	PRMT5↓, E2F-1/NF-κB/NLRP3↑	Male SD rats TAC-induced cardiac hypertrophy model	AC16 cells and HCMs Ang II-induced cell hypertrophy model	(104)
	Sema4D/MAPK/NF-κB/NLRP3↑	Male C57BL/6 mice TAC-induced cardiac hypertrophy model	NRCMs Ang II-induced cell hypertrophy model	(105)
	NLRP3↑, Calcineurin↑, MAPK↑	Male C57BL/6J mice TAC-induced pathological cardiac remodeling model	-	(94)
	ROS/NLRP3/caspase-1↑	Male F344 rats SiNPs intratracheal instillation-induced pathological cardiac hypertrophy model	CMs and AC16 Cells SiNPs-simulated hypertrophy model	(106)
	SNO-MLP/TLR3/RIP3/NF-κB/NLRP3↑	a. Male SHRs, WKY rats, C57BL/6 WT mice TAC coarctation-induced pathological cardiac remodeling model b. Myocardial samples from patients undergoing heart valve replacement surgery	NRCMs Ang II or phenylephrine-induced cell hypertrophy model	(107)
Inhibite angiogenesis	ROS/TXNIP/NLRP3↑	Male C57BL/6 mice LADCA ligation and release-induced MI/R model	NMCMs and CMECs Hypoxia/hypoglycemic and normoxia/normal-glucose-induced cell damage model	(10)
	MicroRNA-495↓, NLRP3↑	Male C57BL/6 mice LADCA ligation and release-induced MI/R model	CMECs (from MI/R mice)	(111)
Disturb cardiac electrical signal conduction	NLRP3↑	a. Blood and left and right ventricular myocardial tissue samples from healthy volunteers and HF patients b. Male Dsg2 gene mutation and WT mice Knockout of the Dsg2 Gene in Cardiomyocytes-induced arrhythmogenic right ventricular cardiomyopathy model	-	(6)
	NLRP3↑	Male C57BL/6 mice TAC-induced HF model	-	(115)
	NLRP3↑	WT and MD1-KO mice Uninephrectomy combined with d-aldosterone perfusion and high-salt diet-induced HF model	-	(117)
	P2X7R/NLRP3↑	Male SD rats LADCA ligation-induced HF model	-	(118)
	SOX2-OT/microRNA-2355-3p/NLRP3↑	Male SPF SD rats Aortic coarctation and constant current stimulation of left carotid sympathetic nerve node-induced HF-VA model	-	(116)
	NLRP3↑, CaMKII↑	Male Dahl salt-sensitive rats High-salt diet-induced HF model	-	(119)
	P2X7R/NLRP3/IL-1β↑	Male SD rats LCA ligation-induced AMI model	Macrophages a. LPS and IFN‐γ-induced M1 macrophage polarization model b. IL-4-induced M2 macrophage polarization model	(120)
	Camk2n1↓, CaMKIIδ/p38 MAPK/JNK/NLRP3↑	Male WT and Camk2n1-KO mice LADCA ligation-induced MI model	MCFs and NMVMs Hypoxia-induced cell damage model	(121)
	NLRP3/IL-1β/p38 MAPK↑	Male SD rats LADCA ligation-induced MI model	-	(123)
	NLRP3/caspase-1/IL-1β/p38↑	Male SD rats LADCA ligation-induced MI model	H9c2 cells Hypoxia-induced cell damage model	(122)
Disturb cardiac energy metabolism	NLRP3↑, CD36↓, CPT1β↓, GLUT4↓, p-PDH↑, AKT↑, AMPKα↓	Male C57BL/6J mice High-fat diet and TAC-induced HF model	-	(7)
	NLRP3↑, RISK↓	-	Isolated hearts (from male Wistar rats) Langendorff perfusion-induced MI/R model	(127)
	NLRP3↑	Male C57BL/6J WT and NLRP3-KO mice Ang II osmotic minipump-induced cardiomyopathy model	-	(125)
	ROS/NF-kB/TXNIP/NLRP3↑	SD rats High-fat diet and streptozotocin intraperitoneal injection-induced diabetic cardiomyopathy model	H9c2 cells High glucose-induced cell damage model	(126)
Exacerbate cardiomyocyte apoptosis	MicroRNA-30a-5p↑, SIRT1↓, NF-κB/NLRP3↑	Male SD rats Aortic coarctation-induced HF model	-	(8)
	CaSR/NLRP3↑	a. Blood samples from healthy volunteers and AMI patients b. Male Wistar rats Coronary artery ligation-induced AMI model	-	(132)
	ZNF561-AS1/microRNA-223-3p/NLRP3↑	Male Kunming mice MI model	HCMs Hypoxic-induced cell damage model	(133)
	NLRP3/caspase-1↑	Male SD rats LADCA ligation and release-induced MI/R model	H9c2 cells H/R-induced cell damage model	(134)
	lncRNA HULC↓, microRNA-377-5p/NLRP3/caspase-1/IL-1β↑	Neonatal SD rats LADCA ligation and release-induced MI/R model	H9c2 cells H/R-induced cell damage model	(135)
	STING/IRF3/NLRP3↑	Male C57/B6 mice LPS intraperitoneal injectione-induced cardiomyopathy model	NRCMs and H9c2 cells LPS-induced cell inflammation model	(136)

↑ indicates activation; ↓ indicates inhibition.

### 3.1 Exacerbate myocardial inflammatory injury

An appropriate inflammatory response serves as a protective mechanism that eliminates harmful stimuli and repairs damaged tissues (57). However, excessive or prolonged inflammation escalates the risk of cardiac dysfunction and adverse cardiac remodeling (57, 58).

In the cardiac tissues of HF rats, increased NLRP3-positive spots, caspase-1 shear activation, and elevated levels of mature IL-1β were accompanied by a heightened inflammatory response (59, 60). These findings suggest that the NLRP3 inflammasome plays a contributory role in the development of myocardial inflammation in HF (59, 60). During acute myocardial infarction (AMI), dying heart myocytes initiate the assembly of the NLRP3 inflammasome by activating P2X7R via ATP release (61). This process amplifies cardiac inflammation, leads to further loss of functional myocardium, and even results in HF (61). Following myocardial infarction (MI), myocardial injury triggers the activation of the NLRP3 inflammasome, which exacerbates the myocardial inflammatory response, leading to enlargement of the infarct and worsening of cardiac dysfunction (62). Nicorandil pretreatment decreased NLRP3 inflammasome activation by inhibiting the TLR4/myeloid differentiation primary response protein 88 (MyD88)/NF-κB pathway, thereby alleviating the detrimental effects of MI on the heart (62). Activation of the NLRP3 inflammasome plays a crucial role in promoting myocardial ischemia/reperfusion (MI/R) injury (63). MI/R injury results in decreased microRNA-148a expression in myocardial cells, which increases the expression of thioredoxin-interacting protein (TXNIP) (64). Subsequently, TXNIP activates the TLR4/NF-κB/NLRP3 signaling pathway, promoting the release of inflammatory factors IL-1β and IL-18, thereby increasing inflammatory cell death in myocardial cells, leading to more extensive myocardial damage and worsening of cardiac function (64). Furthermore, studies have revealed that signaling pathways including adenosine 5′-monophosphate (AMP)-activated protein kinase (AMPK) (65), silent information regulator of transcription (SIRT) 1/serine/threonine protein kinase B (Akt)/pyruvate dehydrogenase (PDH)/ROS (66), and E3 ubiquitin ligase membrane-associated RING finger protein 2 (MARCH2)/phosphoglycerate mutase 5 (PGAM5)/mitochondrial anti-viral-signaling protein (MAVS) (67) contribute to the exacerbation of cardiac inflammatory injury by activating the NLRP3 inflammasome, thereby exacerbating adverse cardiac outcomes caused by MI/R.

Activation of the NLRP3 inflammasome is a pivotal factor contributing to increased inflammatory damage in the non-ischemic myocardium. Activation of the NLRP3 inflammasome led to increased cardiomyocyte pyroptosis and reduced proliferative capacity in the doxorubicin (DOX)-induced HF model, collectively exacerbating the pathogenic progression of HF (68, 69). Mechanistically, activation of the TLR4/MyD88/NF-κB signaling pathway serves as an upstream event that triggers the activation of the NLRP3 inflammasome, thereby exacerbating cardiomyocyte pyroptosis and myocardial inflammation in DOX-induced HF (70, 71). Myocardial tissues from patients with dilated cardiomyopathy (DCM) exhibit aberrant NLRP3 inflammasome activation and pronounced pyroptosis, which are correlated with diminished cardiac function (72). In a DOX-induced DCM mouse model, DOX triggered the hyperactivation of the NLRP3 inflammasome by upregulating NOX1 and NOX4 expression and activating dynamin-related protein 1 (Drp1)-dependent mitochondrial fragmentation (72). This process exacerbates cardiomyocyte pyroptosis and contributes to the progression of cardiac dysfunction (72). Macrophages play a pivotal role in the regulation of cardiac inflammation (73). In the HF state, the activation of the NLRP3 inflammasome in myocardial tissue promotes macrophage infiltration into the heart (74). Mechanistic study has demonstrated that cardiomyocytes activate NLRP3 through the calmodulin-regulated kinase δ (CaMKIIδ) signaling pathway, promoting the release of pro-inflammatory cytokines IL-1β, IL-18, and IL-6, as well as the production of monocyte chemotactic protein-1 (MCP-1) and macrophage inflammatory protein 1α (75). These factors synergistically promote macrophage migration to myocardial tissue, thereby further amplifying cardiac inflammation (75). Furthermore, activation of myocardium-specific CaMKIIδ can also mediate the activation of the NLRP3 inflammasome through the NF-κB pathway, leading to increased macrophage recruitment to the damaged myocardium and exacerbating the cardiac inflammatory cascade (76). Interestingly, macrophages demonstrate two pro-inflammatory effects, pyroptosis and pro-inflammatory polarization, upon recruitment to the heart (77–79). In pulmonary arterial hypertension (PAH)-induced right ventricular failure, there was a significant increase in the number of macrophages within the right ventricle, accompanied by an elevated expression of the NLRP3 inflammasome in these macrophages (78). The elevated expression of the NLRP3 inflammasome not only promoted macrophage pyroptosis, but also drove macrophages toward a pro-inflammatory M1-type phenotype (78). This shift exacerbated the inflammatory response in the right ventricle, contributing to further deterioration of right ventricular dysfunction (78). Additionally, cardiomyocyte NLRP3-dependent pyroptosis further stimulates macrophage polarization toward a pro-inflammatory M1 phenotype in myocardial tissues through the release of pro-inflammatory cytokines and MCP-1 (79). Similarly, in sepsis-induced cardiac inflammatory injury and dysfunction, macrophage pyroptosis in cardiac tissues, along with the polarization of Ly6Chigh macrophages, is positively regulated by NLRP3 complex activation (77). These studies confirm that activation of the NLRP3 inflammasome exacerbates the cardiac inflammatory cascade by promoting macrophage recruitment to the heart and stimulating macrophage pyroptosis and pro-inflammatory polarization.

### 3.2 Aggravate adverse myocardial fibrosis

Myocardial fibrosis is characterized by abnormal proliferation and differentiation of cardiac fibroblasts (CFs) and excessive accumulation and abnormal distribution of the extracellular matrix (ECM). Myocardial fibrosis is a critical reparative response aimed at maintaining cardiac integrity after myocardial injury (80). However, excessive myocardial fibrosis results in diminished myocardial compliance and cardiac diastolic and systolic dysfunction, serving as a pivotal pathological foundation for the onset and progression of HF (81, 82).

Myofibroblasts play a crucial role as mediator cells in the progression of myocardial fibrosis (83). They induce cardiac fibrous scar formation and dysfunction by synthesizing significant quantities of ECM and collagen, secreting pro-fibrotic cytokines, and expressing α-smooth muscle actin (α-SMA) (83). IL-1β was identified as a key mediator in promoting the proliferation and differentiation of CFs into myofibroblasts, indicating that NLRP3 inflammasome activation is an important factor mediating the progression of myocardial fibrosis (84, 85). MI leads to a notable upregulation in the expression of fibrotic markers in myocardial tissues, including collagen I, collagen III, and α-SMA (86). Importantly, the degree of NLRP3 inflammasome activation is positively correlated with the severity of myocardial fibrosis (86). Myocardial ischemia triggered the activation of the TLR4/MyD88/NF-κB signaling pathway, which facilitated the assembly and activation of the NLRP3 inflammasome, thereby exacerbating cardiac inflammation (87). The progression of inflammation enhances fibrosis, resulting in increased cardiac stiffness and reduced cardiac pumping function (87). The expression of calcium-sensitive receptor (CaSR) is elevated in myocardial tissue following MI (88). CaSR exacerbates both inflammation and fibrosis post-MI by activating the autophagy/NLRP3 inflammasome pathway (88).

In pressure overload-induced HF, the activation of the NLRP3 inflammasome was identified as a critical factor driving the progression of myocardial fibrosis (89). Mechanistically, chronic stress overload initiates the activation of the NF-κB/NLRP3 inflammasome pathway (90). This pathway amplifies the aberrant activation of cardiac fibroblasts and promotes the over-synthesis of collagen associated with fibrosis, thereby fueling the adverse progression of cardiac fibrosis (90). Administration of angiotensin II (Ang II) induced myocardial fibrosis in mice, as indicated by the excessive deposition of collagen fibers, elevated expression levels of transforming growth factor-β (TGF-β) and connective tissue growth factor, along with NLRP3 inflammasome activation in cardiac tissues and heightened IL-1β secretion (85). Notably, treatment with MCC950 successfully reversed these pathological alterations (85). Inositol-requiring enzyme 1α (IRE1α) acted as a sensor of endoplasmic reticulum stress, capable of triggering NLRP3 inflammasome activation, thereby exacerbating the progression of myocardial fibrosis (91). The endogenous cardiovascular protective peptide, intermedin_1-53_ (IMD_1-53_), had the ability to reduce the expression of IRE1α through the activation of the cyclic adenosine monophosphate (cAMP)/protein kinase A (PKA) pathway, leading to the inhibition of NLRP3 inflammasome activation and mitigation of Ang II-induced cardiac fibrosis (91). Reduced expression of C1q/TNF-related protein-3 (CTRP3) in myocardial tissue is linked to the advancement of cardiac fibrosis (92). The restoration of CTRP3 expression ameliorated Ang II-induced myocardial fibrosis by inhibiting the P2X7R/NLRP3 inflammasome pathway to reduce α-SMA, collagen I/III, and matrix metallopeptidase (MMP) 2/9 expression (92).

TGF-β is a central signaling pathway in the promotion of fibrosis. In HF, activation of the NLRP3 inflammasome is considered a significant factor in the upregulation of TGF-β gene expression in the cardiac tissue (93). Specifically, the activated NLRP3 inflammasome promotes the advancement of cardiac fibrosis by triggering the TGF-β/Smad4 signaling pathway to enhance the expression levels of collagen type I, collagen type III, MMP-2, MMP-9, and α-SMA (94). Further study has demonstrated that Ang II receptor type 1 (AGTR1) accelerates myocardial fibrosis progression by activating the NLRP3 inflammasome and enhancing the production of TGF-β1 (95). Concurrently, activation of the TLR4 receptor also triggers NLRP3 inflammasome activation, initiating a signaling cascade that enhances the pro-fibrotic effects of the TGF-β1/IL-1β axis, promotes cardiac myofibroblast differentiation, increases interstitial collagen deposition, and ultimately exacerbates fibrosis (96). Additionally, a study revealed the mitochondrial localization of NLRP3 in CFs and demonstrated that NLRP3 is involved in the development of cardiac fibrosis by enhancing mitochondrial ROS production, promoting activation of the TGF-β/R-Smad pathway, and facilitating CF differentiation (97).

### 3.3 Intensify pathological myocardial hypertrophy

Pathological myocardial hypertrophy is an adaptive response of the heart to prolonged pressure or increased volume overload. Nevertheless, as myocardial hypertrophy advances to a certain level, it can exert significant adverse effects on the cardiac structure and function, thereby increasing the risk of HF (98).

In the context of cardiac remodeling, the activation of the NLRP3 inflammasome promotes not only cardiac inflammation and fibrosis but also aggravates pathological myocardial hypertrophy, consequently exacerbating symptoms of HF (99). Elevated levels of G protein-coupled receptor kinase 2 (GRK2) were identified in hypertrophied myocardial tissue (100, 101). Mechanistically, GRK2 promotes the activation of the NLRP3 inflammasome and induces oxidative stress (OS) by downregulating the expression of nuclear factor erythroid-2-related factor 2 (Nrf2), thereby exacerbating isoproterenol (ISO)-induced pathological cardiac hypertrophy (102). The receptor for advanced glycation endproducts (RAGE) participated in Ang II-induced pathological cardiomyocyte hypertrophy by activating the NF-κB/NLRP3/IL-1β pathway (103). Under pressure overload, there is a reduction in protein arginine methyltransferase 5 (PRMT5) expression in hypertrophic myocardial tissues. Low PRMT5 expression triggered the activation of the E2F-1/NF-κB signaling pathway, leading to the activation of the NLRP3 inflammasome that promotes maladaptive cardiac hypertrophy induced by transverse aortic constriction (TAC) or Ang II (104). The overexpression of Sema4D contributed to pressure overload-induced cardiac hypertrophy (105). It promotes the assembly and activation of NLRP3 complexes by activating the mitogen-activated protein kinase (MAPK)/NF-κB signaling pathway, thereby exacerbating TAC-induced pathological cardiac hypertrophy and dysfunction (105). Elevated levels of calcineurin and MAPK phosphorylation were observed in the TAC surgery group of pressure-overloaded mice (94). In contrast, MCC950 ameliorates pathological cardiac hypertrophy and enhances cardiac function by inhibiting calcineurin expression and the MAPK signaling pathway (94). Silica nanoparticles (SiNPs) contributed to the exacerbation of cardiac hypertrophy (106). SiNPs worsen myocardial hypertrophy by inducing cardiomyocyte pyroptosis via activation of the ROS/NLRP3/caspase-1 signaling pathway (106). Transfection of cardiomyocytes with si-NLRP3 or the caspase-1 inhibitor VX-765 limited SiNP-induced pathological cardiac hypertrophy (106). S-nitrosylated muscle LIM protein (SNO-MLP) expression is markedly elevated in patients and animals with myocardial hypertrophy (107). This upregulation primarily facilitated the interaction between TLR3 and receptor-interacting protein kinase 3 (RIP3), thus initiating activation of the NF-κB/NLRP3 inflammasome pathway, ultimately fostering the progression of myocardial hypertrophy (107).

### 3.4 Inhibite angiogenesis

Angiogenesis generates new blood vessels from existing capillaries or capillary post-veins. When the heart is exposed to ischemic and hypoxic stimuli, angiogenesis enhances its blood supply, thereby mitigating damage and preserving cardiac function resulting from ischemia and hypoxia (108, 109). However, the progression of cardiac pathological remodeling inhibits angiogenesis, resulting in decreased microvascular density and ultimately leading to HF (109).

Coronary microvessel rarefaction and decreased blood flow reserve have been identified as the primary drivers of diastolic dysfunction in patients with HF with a preserved ejection fraction (HFpEF) (9). Moreover, decreased cardiac microvascular density is intricately linked to NLRP3 inflammasome activation (110). Phosphorylation of microfibrillar-associated protein 4 (MAP4) downregulates the expression of angiogenic markers, such as CD31, CD34, VEGFA, VEGFR2, ANG2, and TIE2 (110). Mechanistically, MAP4 inhibited angiogenesis via NLRP3 inflammasome activation, leading to reduced cardiac microvessel density (110). Endothelial cells (ECs) serve as primary effector cells in cardiac angiogenesis, and any damage to or aberrant apoptosis of these cells significantly affects their capacity for cardiac angiogenesis. During MI/R injury, microvascular endothelial cells (CMECs) mediated interactions between TXNIP and NLRP3 by generating excessive ROS (10). This action subsequently escalates the activation level of the NLRP3 inflammasome, exacerbating damage to cardiac microvascular endothelial cells (10). In ischemia-reperfused myocardial tissues, there was a reduction in microRNA-495 expression, which facilitates the activation of the NLRP3 inflammasome, worsening inflammatory damage and apoptosis in CMECs (111). Conversely, elevating the expression of microRNA-495 or suppressing the NLRP3 gene decreases apoptosis and enhances the proliferation of CMECs by shifting the cell population from the G0/G1 phase to the S phase (111). This observation implies that the suppression of NLRP3 inflammasome activation may facilitate the repair and angiogenesis of cardiac microvessels. SIRT3 deficiency resulted in diminished expression of hypoxia-inducible factor-2α, VEGF, and angiopoietin-1, leading to decreased angiogenesis and subsequently causing coronary microvessel rarefaction and cardiac diastolic dysfunction (112). Trimethylamine N-oxide (TMAO) induces vascular inflammation by suppressing SIRT3 expression and superoxide dismutase 2 (SOD2) activity in endothelial cells, subsequently triggering mtROS/NLRP3 inflammasome signaling (113). Therefore, SIRT3 deficiency may impede coronary microvascular angiogenesis by activating the NLRP3 inflammasome.

### 3.5 Disturb cardiac electrical signal conduction

Ventricular arrhythmias (VAs) are common triggers and causes of death in HF (114). The cardiac electrical conduction system is crucial for maintaining normal heart function, and conduction abnormalities are the underlying precursors of arrhythmias.

Numerous studies have established that the activation of the NLRP3 inflammasome is a key factor in disrupting the electrical signaling in the heart and inducing malignant arrhythmias, particularly in the context of HF (6, 115–119). The activation of the NLRP3 inflammasome not only enhances myocardial inflammatory responses but also promotes the development of cardiac hypertrophy and fibrosis, creating a pro-arrhythmic environment (115). Additionally, NLRP3 inflammasome activation results in changes to myocyte ion channel functions, including a reduced expression of ion channel proteins such as Kv4.2, KChIP2, and Cav1.2, which affect the timing and morphology of cardiac action potentials and contribute to the development and maintenance of arrhythmias (115). Simultaneously, sympathetic nervous hyperactivity contributes to an increased susceptibility to HF-related ventricular arrhythmias due to NLRP3 inflammasome activation (117, 118). In particular, the activation of the NLRP3 inflammasome exacerbates cardiac sympathetic hyperactivity by promoting the release of the proinflammatory cytokine IL-1β, and this inflammatory-neural interaction results in altered electrophysiological properties of the heart, such as prolongation of the action potential duration and shortening of the effective refractory period, which increases the risk of ventricular arrhythmias (117, 118). In the myocardial tissues of rats with HF-related ventricular arrhythmias (VAs-HF), there was a notable increase in the expression of SOX2-overlapping transcript (SOX2-OT) and NLRP3 (116). Furthermore, silencing of the SOX2-OT gene reduced NLRP3 inflammasome activation levels by regulating microRNA-2355-3p, thus alleviating HF symptoms and diminishing VAs (116). In HFpEF, the activation of the NLRP3 inflammasome facilitates the development of atrial fibrillation through the promotion of atrial fibrosis, by prolonging the atrial action potential duration, increasing the dispersion of action potential duration, and activating inflammation-associated signaling pathways (119). Following MI, P2X7R facilitates the upregulation of nerve growth factor, tyrosine hydroxylase, and growth-associated protein 43 by mediating the activation of the NLRP3/IL-1β pathway, thereby fostering sympathetic sprouting (120). This cascade leads to altered cardiac electrophysiological characteristics and an increased susceptibility to arrhythmias (120). After MI, the expression of Camk2n1 is markedly reduced in the infarct border zone, leading to the activation of the CaMKIIδ/p38 MAPK/C-Jun N-terminal kinase (JNK)/NLRP3 inflammasome signaling pathway (121). This exacerbates myocardial fibrosis and increases the vulnerability to premature ventricular contractions, tachycardia, and ventricular fibrillation (121). Connexin 43 (Cx43) is a key regulator of cardiac electrical signal conduction (122, 123). The activation of the NLRP3 inflammasome within the myocardial infarct zone diminishes the expression of Cx43 in myocardial tissue, resulting in compromised intercellular communication and heightened vulnerability to VAs (122, 123). Conversely, restoring the expression of Cx43 in the heart by inhibiting the NLRP3/IL-1β/p38 MAPK pathway helps enhance cardiac conduction function and decrease the heart’s susceptibility to VAs (122, 123).

### 3.6 Disturb cardiac energy metabolism

The heart, as an organ with high energy and oxygen demands, relies on homeostasis of its energy metabolism as the foundational mechanism for maintaining the stability of the cardiac tissue structure and internal environment (124). The myocardial energy metabolism relies heavily on mitochondrial oxidative phosphorylation. When mitochondria are damaged, myocardial energy substrate utilization is altered, leading to decreased cardiac energy production and lactic acid build-up, which in turn affects cardiac energy metabolism and cardiomyocyte survival and accelerates the malignant progression of HF (124).

A complex interplay exists between NLRP3 inflammasome activation and myocardial energy metabolism disruption. Mitochondrial dysfunction is the trigger for the activation of the NLRP3 inflammasome (36, 37), while the activation of the NLRP3 inflammasome further impairs mitochondrial function and homeostasis (7, 125–127). In an obesity-associated HF model, overactivation of the NLRP3 inflammasome results in an imbalance between cardiac energy supply and demand, as evidenced by decreased fatty acid utilization and increased reliance on glycolysis and glucose oxidation in cardiomyocytes, thereby triggering cardiac metabolic reprogramming (7). This metabolic transition was concomitant with the downregulation of genes associated with mitochondrial energy transfer and respiratory pathways, consequently intensifying the advancement of HF (7). During MI/R injury, the inhibition of the NLRP3 inflammasome activates the reperfusion injury salvage kinase (RISK) pathway, subsequently enhancing the expression of markers associated with mitochondrial biogenesis and energy metabolism, such as mitochondrial transcription factor A, nuclear respiratory factor-1, and mitochondrial creatine kinase (127). These findings suggest an association between disturbed myocardial energy metabolism and the formation of the NLRP3 inflammasome complex during MI/R injury, indicating that inhibition of NLRP3 inflammasome activation contributes to the improvement of cardiac energy metabolism, thereby enhancing the resistance of cardiomyocytes to ischemic and hypoxic injury (127). In the Ang II-induced cardiomyopathy model, increased NLRP3 inflammasome activity was accompanied by decreased mtDNA copy number, reduced ATP synthase activity, increased ROS production, as well as mitochondrial structural alterations, including swelling, disordered matrix organization, and fragmentation (125). The knockdown of the NLRP3 gene mitigated Ang II-induced mitochondrial structural and functional damage, as well as alleviated cardiac dysfunction (125). In rats with diabetic cardiomyopathy, cardiomyocyte mitochondria exhibit swelling and matrix disorders, along with activation of the NLRP3 inflammasome (126). Silencing of the NLRP3 gene aided in restoring mitochondrial structure and reducing glycogenolysis and lipid accumulation in cardiomyocytes, suggesting an enhancement in cardiomyocyte energy metabolism (126).

### 3.7 Exacerbate cardiomyocyte apoptosis

Cardiomyocyte apoptosis is a type of programmed cell death that is genetically regulated (128). Cardiomyocytes, which are primary cardiac cells, are responsible for contraction (129). Excessive apoptosis of cardiomyocytes is a significant contributor to the structural alterations and functional deterioration of the heart. Moreover, it is a crucial driver of HF onset and progression (130, 131).

During the pathological progression of HF, the overactivation of NLRP3 exerts a pro-apoptotic effect on cardiomyocytes (8). Mechanistically, microRNA-30a-5p activates the NF-κB/NLRP3 signaling cascade by targeting SIRT1, thereby exacerbating cardiomyocyte apoptosis (8). CaSR expression is markedly elevated in the neutrophils of patients and rats with AMI (132). This upregulation facilitated NLRP3 inflammasome activation, release of IL-1β through the PLC-IP3 pathway, and calcium release from the endoplasmic reticulum (132). IL-1β interacted with the IL-1 receptor on cardiomyocytes, leading to an increase in Bax expression and caspase-3 cleavage, while decreasing Bcl2 expression, thereby effectively promoting cardiomyocyte apoptosis (132). In the myocardial tissue of MI mice, the expression of the long noncoding RNA zinc finger protein 561 antisense RNA 1 (ZNF561-AS1) is significantly upregulated (133). This upregulation leads to the inhibition of cardiomyocyte proliferation and augmentation of cardiomyocyte apoptosis via activation of the microRNA-223-3p/NLRP3 inflammasome pathway (133). During MI/R injury, activation of the NLRP3 inflammasome results in increased cardiomyocyte apoptosis through the upregulation of Bax protein expression and downregulation of Bcl2 expression (134). In myocardial tissues injured by ischemia reperfusion, the expression of long noncoding RNA highly up-regulated in liver cancer (lncRNA HULC) is downregulated (135). The decrease in lncRNA HULC expression results in heightened microRNA-377-5p activity, triggering the NLRP3/caspase-1/IL-1β signaling pathway (135). This cascade amplifies caspase-3 and cleaved-caspase-3 expression, ultimately worsening cardiomyocyte apoptosis (135). In a mouse model of cardiomyopathy, STING activation triggers the activation of the NLRP3 inflammasome by enhancing the phosphorylation and intranuclear translocation of IRF3 (136). This process elevates the ratios of Bax/Bcl-2 and C-Caspase3/T-Caspase3, leading to an increase in cardiomyocyte apoptosis (136).

## 4 TCM active ingredients in preventing and treating HF by inhibiting the NLRP3 inflammasome

Active ingredients are fundamental to the efficacy of TCM. Existing studies have revealed that active ingredients in TCM exert positive regulatory effects on key pathological processes of HF by inhibiting the NLRP3 inflammasome. In particular, these active ingredients are effective in ameliorating myocardial inflammation, adverse myocardial fibrosis, pathological myocardial hypertrophy, angiogenesis, cardiac electrical signal conduction, cardiac energy metabolism, and reducing abnormal cardiomyocyte apoptosis (**Table 2**). Further analysis revealed that these active ingredients, with the potential to prevent and treat HF, are primarily found in flavonoids and their glycosides, terpenes and their glycosides, phenolic acids, quinones, and phenylpropanoids (**Table 2**).

**Table 2:** Mechanisms of active ingredients in TCM regulating the NLRP3 inflammasome in HF.

Active ingredients		Mechanisms	Effects	Models		References
				*In Vivo*	*In Vitro*	
Flavonoids and their glycosides	Astragaloside IV	NLRP3↓, GDF15↓, CRP↓, IL1RL1↓, MCP-1↓, PDH↑	Alleviate myocardial inflammation, fibrosis and hypertrophy, and improve cardiac energy metabolism	Male C57BL/6N mice High-fat diet and administration of N-ω-Nitro-L-Arginine methyl ester induced-HF model	-	(17)
		ROS/NLRP3/caspase-1/GSDMD↓	Alleviate myocardial inflammation, fibrosis and hypertrophy	Male SPF C57BL/6J mice LADCA ligation-induced MI model	BMDMs LPS-induced cell inflammation model	(137)
		LC3II↑, p62↓, ROS/NLRP3↓	Alleviate myocardial inflammation and hypertrophy	Male SD rats Abdominal aortic constriction-induced cardiac hypertrophy model	RCMs Mechanical stretch-induced cell hypertrophy model	(138)
		SIRT1↑, NLRP3↓	Alleviate myocardial inflammation	Male C57BL/6J mice DOX intraperitoneal injection-induced myocardial toxicity model	H9c2 cells DOX-induced cell toxicity model	(139)
	Phloretin	NLRP3/caspase-1/IL-1β/p38↓	Alleviate myocardial inflammation and fibrosis, and improve electrical signal conduction.	Male SD rats LADCA ligation-induced MI model	H9c2 cells Hypoxia-induced cell damage model	(122)
	Scutellarin	Akt↑, mTORC1/NLRP3↓	Alleviate myocardial inflammation and reduce cardiomyocyte apoptosis	Male SD rats LADCA ligation and release-induced MI/R model	H9c2 cells OGD/R-cell damage model	(140)
	Hydroxylsafflower yellow A	AMPK↑, mTOR/NLRP3↓	Alleviate myocardial inflammation and reduce cardiomyocyte apoptosis	Male SD rats LADCA ligation and release-induced MI/R model	-	(143)
		AMPK↑, NLRP3↓	Alleviate myocardial inflammation, improve energy metabolism, and reduce cardiomyocyte apoptosis	-	H9c2 cells H/R-induced cell damage model	(142)
		NLRP3/caspase-1/GSDMD↓	Alleviate endothelial inflammation	-	HUVECs OGD/R-induced cell damage model	(141)
	Formononetin	ROS/TXNIP/NLRP3↓	Alleviate myocardial inflammation and reduce cardiomyocyte apoptosis	Male SD rats LADCA ligation and release-Induced MI/R model	NRCMs LPS and nigericin-induced cell inflammation model	(144)
	Luteolin	TLR4/NF-κB/NLRP3↓	Alleviate myocardial inflammation	Male SD rats LADCA ligation and release-induced MI/R model	H9c2 cells H/R-induced cell damage model	(145)
		SIRT1↑, NLRP3/NF-κB↓	Alleviate myocardial inflammation and improve electrical signal conduction	Male SD rats LADCA ligation and release-induced MI/R model	-	(146)
	Biochanin A	TLR4/NF-kB/NLRP3↓	Alleviate myocardial inflammation	Male SD rats LADCA ligation and Release-induced MI/R model	-	(147)
	Irisin	NLRP3↓	Alleviate myocardial inflammation, fibrosis and hypertrophy	Male C57BL/6J mice TAC-induced cardiac hypertrophy model	CMs Ang-II-induced cell hypertrophy model	(148)
	Amentoflavone	STING/NLRP3↓	Alleviate myocardial inflammation, fibrosis and hypertrophy, and reduce cardiomyocyte apoptosis	Male C57BL/6J mice DOX intraperitoneal injection-induced myocardial toxicity model	ventricular CMs, MDA-MB-231 cells and MCF-7 cells DOX-induced cell toxicity model	(149)
	Calycosin	SIRT1↑, NLRP3↓, OS↓	Alleviate myocardial inflammation and fibrosis, and reduce cardiomyocyte apoptosis	Male Kunming mice DOX intraperitoneal injection-induced myocardial toxicity model	H9c2 cells DOX-induced cell toxicity model	(150)
Terpenoids and their glycosides	Gentiopicroside	Nrf2↑, NLRP3↓	Alleviate myocardial inflammation and reduce cardiomyocyte apoptosis	SD rats LADCA ligation-induced AMI model	H9c2 cells H/R-induced cell damage model	(151)
	Celastrol	NLRP3↓	Alleviate myocardial inflammation and fibrosis, improve electrical signal conduction, and reduce cardiomyocyte apoptosis	Male SD rats LCA ligation induced-HF model	H9c2 cells Hypoxia-induced cell damage model	(16)
		NLRP3↓	Alleviate myocardial inflammation and fibrosis	Male SD rats LADCA ligation-induced MI model	NRCFs LPS and ATP-induced cell inflammation model	(152)
	Muscone	NLRP3/IL-1β/p38 MAPK↓	Alleviate myocardial inflammation and fibrosis, and improve electrical signal conduction	Male SD rats LADCA ligation-induced MI model	-	(123)
		ROS↓, NF-κB↓, NLRP3 ↓	Alleviate myocardial inflammation	Male C57BL/6J mice LADCA ligation-induced MI model	BMDMs Starvation and LPS-induced cell inflammation model	(153)
	Oridonin	NLRP3↓	Alleviate myocardial inflammation and fibrosis	Male C57BL/6 mice LCA ligation-induced MI model	BMDMs LPS-induced cell inflammation model	(154)
		OS↓, NLRP3↓	Alleviate myocardial inflammation	Male C57BL/6 mice LADCA ligation and release-induced MI/R model	-	(155)
	Sweroside	CaMKIIδ/ROS/NF-κB/NLRP3↓	Alleviate myocardial inflammation, fibrosis and hypertrophy	Male C57BL/6 N mice TAC and Ang II perfusion induced-HF model	H9c2 cells, AC16 cells and NRCMs Ang II-induced cell hypertrophy model	(156)
		Keap1↓, Nrf2↑, OS↓, NLRP3↓	Alleviate myocardial inflammation	**-**	a. Myocardial tissue (from male Wistar rats) O_2_-saturated Krebs–Henseleit solution-induced MI/R model b. H9c2 cells H/R-induced cell damage model	(157)
	Geniposide	AMPK↑, ROS/TXNIP/NLRP3↓	Alleviate myocardial inflammation and improve cardiac energy metabolism	Male C57BL/6J mice LADCA ligation and release-induced MI/R model	NRVMs and H9c2 cells H/R-induced cell damage model	(158)
	Loganin	GLP-1R↑, NLRP3↓	Alleviate myocardial inflammation and reduce cardiomyocyte apoptosis	Male SD rats LADCA ligation and release-induced MI/R model	H9c2 cells OGD/R-induced cell damage model	(159)
	Artemisinin	NLRP3↓, autophagy↓, OS↓	Alleviate myocardial inflammation and fibrosis, improve mitochondrial function, and reduce cardiomyocyte apoptosis	Male SD rats LCA ligation and release-induced MI/R model	-	(160)
	Betulin	SIRT1↑, NLRP3/NF-κB↓	Alleviate myocardial inflammation and improve electrical signal conduction	Wistar rats LADCA ligation and release-induced MI/R model	-	(161)
	Triptolide	NLRP3/TGF-β1/Smad3↓	Alleviate myocardial inflammation, fibrosis and hypertrophy	Male C57/BL6 mice TAC-induced cardiac remodeling model	-	(162)
		MyD88↓, JNK↓, ERK1/2↓, NLRP3/TGF-β1/Smad↓	Alleviate myocardial inflammation and fibrosis	male C57 WT, NLRP3-KO mice ISO subcutaneous injection-induced myocardial fibrosis model	CFs Ang II-simulated fibrosis model	(163)
	Ginsenoside Rg3	SIRT1↑, NF-κB/NLRP3↓, OS↓	Alleviate myocardial inflammation, fibrosis and hypertrophy	SD rats TAC-induced cardiac hypertrophy model	AC16 cells and HCMs Ang II-induced cell hypertrophy model	(164)
	Ginsenoside Rb1	DUSP-1/TMBIM-6/VDAC1↑, NLRP3↓	Alleviate myocardial inflammation, fibrosis and hypertrophy, improve cardiac energy metabolism, and reduce cardiomyocyte apoptosis	Male C57BL/6 WT, DUSP-1-KO, DUSP-1-KI, VDAC1-KO and VDAC1-KI mice TAC-induced HF model	Ventricular myocytes H/R-induced cell damage model	(165)
		NLRP3↓, calcium overload ↓	Alleviate myocardial inflammation, improve electrical signal conduction and mitochondrial structure, and reduce cardiomyocyte apoptosis	Male SD rats Aconitine gavage administration-induced cardiac toxicity model	HiPSC-CMs and ARVMs Aconitine-induced cell toxicity model	(166)
	Ginsenoside Rg1	TLR4/NF-kB/NLRP3↓	Alleviate myocardial inflammation and reduce cardiomyocyte apoptosis	Male C57BL/6J mice LPS intraperitoneal injection-induced cardiac dysfunction model	NRCMs LPS-induced cell inflammation model	(167)
	Shikonin	SIRT1↑, NLRP3↓	Alleviate myocardial inflammation and reduce cardiomyocyte apoptosis	Male C57BL/6J mice LPS intraperitoneal injection-induced cardiac dysfunction model	H9c2 cells LPS-induced cell inflammation model	(168)
Phenolic acids	resveratrol	SIRT1↑, p53↓, NLRP3↓	Alleviate myocardial inflammation and fibrosis, and reduce cardiomyocyte apoptosis	Male C57BL/6J mice a. LCA ligation-induced MI model b. LADCA ligation and release-induced MI/R model	a. NRCMs and CFs H/R-induced cell damage model b. Macrophages LPS-induced cell inflammation model	(170)
		Akt1/NLRP3↓	Alleviate myocardial inflammation	Male C57BL/6J mice ISO subcutaneous injection-induced acute sympathetic stress model	NMCMs ISO-simulated acute sympathetic stress model	(171)
	Salvianolic acid B	SIRT1/AMPK/PGC-1α↑, NLRP3↓	Alleviate myocardial inflammation, improve cardiac energy metabolism, and reduce cardiomyocyte apoptosis	Male SD rats LADCA ligation-induced MI model	H9c2 cells Hypoxia-induced cell damage model	(172)
		Mitophagy↑, NLRP3↓	Alleviate myocardial inflammation, improve mitochondrial function, and reduce cardiomyocyte apoptosis	Male SD rats ISO subcutaneous injection-induced acute myocardial ischemia model	H9c2 cells LPS and ATP-induced cell inflammation model	(173)
	Cichoric acid	HK1/NLRP3↓	Alleviate myocardial inflammation and fibrosis, improve cardiac energy metabolism, and reduce cardiomyocyte apoptosis	Male Kunming mice ISO subcutaneous injection-induced myocardial fibrosis model	**-**	(174)
	Curcumin	Akt/mTOR↑, NLRP3↓, Autophagy↓	Alleviate myocardial inflammation, improve mitochondrial structure, and reduce cardiomyocyte apoptosis	Male Kunming mice DOX intraperitoneal injection-induced myocardial toxicity model	H9c2 cells DOX-induced cell toxicity model	(175)
	Carvacrol	NLRP3/caspase-1/GSDMD↓, OS↓, Autophagy↑	Alleviate myocardial inflammation	Male Balb/C mice LPS intraperitoneal injection-induced cardiac dysfunction model	H9c2 cells LPS-induced cell inflammation model	(176)
Quinones	Tanshinone IIA	TLR4/NF-κB p65/NLRP3↓	Alleviate myocardial inflammation and fibrosis, improve mitochondrial structure, and reduce cardiomyocyte apoptosis	Male SD rats LADCA ligation-induced AMI model	H9c2 cells H/R-induced cell damage model	(177)
	Salvianolate	TGF-β1/Smad2/3↓,TXNIP/NLRP3↓	Alleviate myocardial inflammation and fibrosis, and improve electrical signal conduction	Male SPF SD rats LADCA ligation-induced MI model	-	(178)
	Emodin	TLR4/MyD88/NF-κB/NLRP3↓	Alleviate myocardial inflammation	Male SD rats LADCA ligation and release-induced MI/R model	NRCMs H/R-induced cell damage model	(179)
	Emodin	NLRP3↓	Alleviate myocardial inflammation	Male C57BL/6 mice LPS intraperitoneal injection-induced cardiac dysfunction model	H9c2 cells and CMs LPS-induced cell inflammation model	(180)
	Sodium tanshinone IIA sulfonate	Autophagy↑, NLRP3↓	Alleviate myocardial inflammation and reduce cardiomyocyte apoptosis	Male C57BL/6 WT mice LPS intraperitoneal injection-induced cardiac dysfunction model	-	(181)
Phenylpropanoids	Beta-asarone	NLRP3↓	Alleviate myocardial inflammation	Male SD rats LADCA ligation and release-induced MI/R model	-	(182)
	Cinnamaldehyde	NLRP3↓	Alleviate myocardial inflammation and reduce cardiomyocyte apoptosis	Male SD rats LADCA ligation and release-induced MI/R model	-	(183)
	Aesculin	Akt↑, GSK3β↑, NF-κB/NLRP3↓	Alleviate myocardial inflammation, improve electrical signal conduction and mitochondrial function, and reduce cardiomyocyte apoptosis	Male SD rats LADCA ligation and release-induced MI/R model	NRCMs OGD/R-induced cell damage model	(184)
	Cinnamic acid	NLRP3/caspase-1/GSDMD↓	Alleviate myocardial inflammation, improve mitochondrial structure, and reduce cardiomyocyte apoptosis	Male SPF SD rats LADCA ligation and release-induced MI/R model	-	(185)
Others	Gastrodin	NLRP3↓	Alleviate myocardial inflammation, stimulate angiogenesis, and reduce cardiomyocyte apoptosis	Male C57BL/6J mice LADCA ligation and release-induced MI/R model	HCMECs H/R-induced cell damage model	(186)
	Panaxynol	HMGB1/TLR4/NF-κB/NLRP3↓	Alleviate myocardial inflammation and reduce cardiomyocyte apoptosis	Male mice LADCA ligation and release-induced MI/R model	NRVMs H/R-induced cell damage model	(187)
	ethyl acetate extract of *Cinnamomi Ramulus*	NLRP3↓	Alleviate myocardial inflammation	Male SD rats LADCA ligation and release-induced MI/R model	-	(188)

↑ indicates activation; ↓ indicates inhibition.

### 4.1 Flavonoids and their glycosides

Astragaloside IV (AS-IV) demonstrates significant therapeutic potential for HFpEF (17). Specifically, AS-IV intervention markedly decreased NLRP3, IL-1β, and caspase-1 levels in the myocardium of HFpEF mice, with this reduction of biomarkers significantly linked to the amelioration of myocardial inflammation and enhancement of cardiac function (17). Additionally, AS-IV exerted a beneficial effect on maintaining cardiac metabolic homeostasis in HFpEF by optimizing cardiac glycolipid metabolism, enhancing mitochondrial function, and regulating energy metabolic pathways (17). AS-IV also effectively alleviated cardiac remodeling caused by MI (137). By inhibiting the ROS/caspase-1/GSDMD signaling pathway, As-IV reduces cardiomyocyte pyroptosis and lowers the expression levels of collagen I, collagen III, α-SMA, and fibronectin (137). This process effectively reduces post-MI cardiac fibrosis and hypertrophy, consequently enhancing the heart function (137). As-IV exerts protective effects against pressure overload-induced cardiac dysfunction (138). Through the upregulation of LC3II levels and inhibition of p62 expression, As-IV activated autophagy, subsequently inhibiting the ROS/NLRP3 inflammasome pathway and reducing the expression levels of IL-1β and IL-18 (138). This action effectively alleviates pressure overload-induced myocardial hypertrophy (138). In addition, As-IV mitigates DOX-induced myocardial toxicity (139). It exerts cardioprotective effects by reversing the DOX-induced downregulation of SIRT1 protein expression, upregulation of NLRP3 expression, and reduction in cardiomyocyte pyroptosis (139). Phloretin mitigates the electrical remodeling process in the heart post-MI (122). By inhibiting the NLRP3/caspase-1/IL-1β pathway, it diminished p38 phosphorylation, facilitating the restoration of Cx43 expression and mitigating cardiac electrical remodeling post-MI, consequently lowering cardiac susceptibility to VAs and the occurrence of HF (122). Furthermore, Phloretin also decreased the expression of fibrotic markers including collagen 1, collagen 3, TGF-β, and α-SMA post-MI by suppressing inflammatory responses orchestrated by NLRP3 inflammasome activation, consequently alleviating detrimental cardiac remodeling (122). The cardioprotective effects of scutellarin are mediated by its regulation of the Akt/mTORC1/NLRP3 signaling pathway (140). More precisely, scutellarin inhibits mTORC phosphorylation by upregulating Akt expression (140). This action subsequently diminishes the activation of the NLRP3 inflammasome, thus mitigating inflammatory injury and dysfunction in the heart induced by MI/R (140). Hydroxylsafflower yellow A (HSYA) was recognized for its ability to mitigate myocardial ischemia and hypoxic injury (141–143). In MI/R injury, HSYA suppressed the NLRP3 inflammasome by modulating the AMPK/mTOR signaling pathway, thereby reducing myocardial infarct size and decreasing cardiomyocyte apoptosis, ultimately improving heart function (143). In an H/R-induced H9c2 cell study, the AMPK inhibitor compound C nullified the suppressive impact of HSYA on NLRP3 inflammasome activation, as demonstrated by elevated levels of NLRP3, caspase-1, and IL-1β expression (142). This observation further corroborates that the inhibition of the AMPK/NLRP3 inflammasome signaling pathway is an important mechanism in the anti-MI/R injury effect of HSYA (142). In a study on oxygen-glucose deprivation/reoxygenation (OGD/R)-induced HUVECs, NLRP3 inflammasome-mediated pyroptosis was heightened (141). Treatment with HSYA mitigated pyroptosis by inhibiting the NLRP3/caspase-1/GSDMD pathway, thereby mitigating inflammatory damage to HUVECs resulting from OGD/R (141). Formononetin can alleviate MI/R injury (144). It restricts the activation of the NLRP3 inflammasome by diminishing the release of ROS, suppressing the expression of TXNIP, and attenuating the interaction between TXNIP and NLRP3, thereby decreasing the secretion of proinflammatory factors and cardiomyocyte apoptosis (144). Luteolin similarly demonstrated the potential to alleviate MI/R injury, and this protective attribute was associated with its suppression of the TLR4/NF-κB/NLRP3 inflammasome pathway (145). Luteolin downregulates the expression of TLR4, MyD88, and NF-κB in a dose-dependent manner to inhibit NLRP3 inflammasome activation, consequently diminishing myocardial infarct size and enhancing left ventricular function (145). Intriguingly, another study identified the SIRT1/NLRP3/NF-κB signaling pathway as the primary regulatory mechanism by which luteolin alleviates MI/R damage (146). These findings suggest that luteolin may exert cardioprotective effects by inhibiting the NLRP3 inflammasome through multiple molecular signaling pathways. Biochanin A alleviates the cardiac inflammatory response and reduces the infarcted myocardial area resulting from MI/R (147). Its cardioprotective effect was intricately linked to its inhibition of the TLR4/NF-κB/NLRP3 signaling pathway (147). By inhibiting NLRP3 inflammasome activation, irisin effectively restrained the expression of GSDMD-N and IL-1β, thereby mitigating the detrimental effects of pressure overload on the heart such as myocardial inflammation, fibrosis, and hypertrophy (148). By inhibiting the STING/NLRP3 signaling pathway, amentoflavone mitigates cardiomyocyte pyroptosis and cardiac inflammation, consequently ameliorating DOX-induced heart damage and functional impairment (149). Calycosin also shows promise for the treatment of myocardial toxicity (150). Mechanistically, it inhibited NLRP3 inflammasome activation by upregulating SIRT1 expression, thereby reducing cardiac inflammatory infiltration, myocardial fibrosis, and cardiomyocyte apoptosis, ultimately mitigating DOX-induced cardiac injury (150).

### 4.2 Terpenoids and their glycosides

AMI triggered intense inflammatory responses and oxidative stress (OS) (151). Gentiopicroside mitigates cardiac inflammatory responses, OS, and cardiomyocyte apoptosis induced by AMI by regulating the Nrf2/NLRP3 signaling pathway, thereby safeguarding cardiac function (151). In the pathological progression of chronic HF, Celastrol improves cardiac electrophysiological stability, upregulates Cx43 and ion channel expression, and reduces myocardial fibrosis and inflammatory responses by inhibiting the NLRP3/caspase-1/IL-1β signaling pathway, ultimately reducing susceptibility to ventricular fibrillation (16). Following MI, a notable increase was observed in macrophage and neutrophil infiltration of myocardial tissues alongside a significant upregulation in the expression of pro-fibrotic proteins such as collagen I, collagen III, and α-SMA (152). Celastrol mitigates these pathological alterations by inhibiting the NLRP3 inflammasome (152). Muscone exhibits a promising therapeutic potential against MI (123). It diminishes ventricular inflammation and fibrosis, while decreasing vulnerability to VAs via the upregulation of Cx43 expression in the infarct border zone (123). These effects were associated with its inhibitory impact on the NLRP3/IL-1β/p38 MAPK pathway (123). Furthermore, Muscone mitigated the macrophage-driven cardiac inflammatory response by suppressing NF-κB expression and NLRP3 inflammasome activation in myocardial macrophages, leading to enhanced cardiac function and increased survival rates in mice post-MI (153). Oridonin can alleviate cardiac remodeling post-MI (154). By inhibiting the NLRP3 inflammasome, it reduced the expression of fibrosis markers, including collagen-I, collagen-III, collagen-IV, and α-SMA, thereby alleviating myocardial fibrosis and cardiac dysfunction following MI (154). Moreover, pretreatment with oridonin suppressed the overactivation of OS and NLRP3 inflammasome, consequently mitigating cardiac pathological alterations induced by ischemia reperfusion, including the alleviation of myocardial inflammatory damage and reduction of infarct size (155). Sweroside inhibits the ROS-mediated NF-κB/NLRP3 inflammasome pathway in cardiomyocytes by directly binding to CaMKIIδ, alleviating myocardial inflammation and adverse cardiac remodeling, thereby improving HF induced by pressure overload (156). Sweroside also exerts protective effects on ischemia reperfusion myocardium (157). Its intervention alleviates myocardial inflammatory damage and reduces the size of the infarcted area, helping to alleviate cardiac dysfunction caused by MI/R (157). This effect is primarily due to the inhibition of NLRP3 inflammasome-mediated pyroptosis (157). Geniposide has therapeutic potential for alleviating MI/R injury (158). It inhibits the ROS/TXNIP/NLRP3 inflammasome pathway by activating the AMPK signaling pathway (158). This process efficiently suppresses cardiac inflammation, enhances myocardial energy metabolism, and ultimately reduces the damage inflicted on the myocardium by ischemia reperfusion (158). The glucagon-like peptide-1 receptor (GLP-1R)/NLRP3 pathway plays a pivotal role in mediating the cardioprotective effects of loganins (159). MI/R induces a notable decline in GLP-1R expression within the myocardial tissue, which promotes the formation of the NLRP3 inflammasome and pyroptosis, exacerbating myocardial damage and cardiomyocyte apoptosis (159). Conversely, treatment with loganin alleviates these pathological changes (159). Artemisinin pretreatment mitigates MI/R-induced myocardial inflammation, cardiomyocyte apoptosis, and myocardial fibrosis primarily by inhibiting the NLRP3 inflammasome (160). Betulin attenuated the cardiac inflammatory response, decreased myocardial infarct size, and enhanced cardiac electrical signaling by modulating the SIRT1/NLRP3/NF-κB signaling pathway. This action ultimately helps mitigate the cardiac pathological damage induced by MI/R (161). Tretinoin has proven advantageous in alleviating negative cardiac repercussions induced by pressure overload (162). Mechanistically, tretinoin impeded the TGF-β1/Smad3 pathway by dampening the activation of the NLRP3 inflammasome, which in turn attenuated TAC-induced myocardial fibrosis and hypertrophy and improved cardiac function (162). Another study demonstrated that the mechanism by which tretinoin mitigates myocardial fibrosis involves the inhibition of the NLRP3 inflammasome. By diminishing MyD88-mediated JNK and ERK1/2 activity, tretinoin suppressed the NLRP3 inflammasome, subsequently inhibiting the TGF-β1/Smad signaling pathway (163). This cascade of events aids in reducing ECM deposition caused by pressure overload, thus exerting an anti-myocardial fibrotic effect (163). Ginsenoside Rg3 inhibited pathological myocardial hypertrophy induced by pressure overload (164). It achieved its anti-inflammatory and antioxidant effects by regulating the SIRT1/NF-κB/NLRP3 inflammasome signaling pathway, thereby reducing myocardial fibrosis and hypertrophy (164). Ginsenoside Rb1 attenuated HF induced by MI/R injury by targeting the DUSP-1/TMBIM-6/VDAC1 pathway, regulating intestinal microbiota homeostasis and the equilibrium of the mitochondrial quality control network, as well as suppressing the NLRP3-mediated inflammatory response and pyroptosis (165). Cardiac injury is a common adverse effect of aconitine. Following aconitine intervention, there is an increase in the expression of NLRP3-dependent pyroptosis-related proteins in myocardial tissue, accompanied by disruptions in electrophysiology, significant myocardial apoptosis, and cardiac dysfunction (166). Ginsenoside Rb1 effectively ameliorated aconitine-induced cardiac pathological alterations (167). Ginsenoside Rg1 effectively mitigated LPS-induced cardiotoxicity by reducing cardiac inflammation and cardiomyocyte apoptosis (167). This was achieved by lowering the Bax/Bcl2 ratio and the quantity of TUNEL-positive cells in myocardial tissues via the inhibition of the TLR4/NF-κB/NLRP3 pathway (167). Shikonin also attenuates LPS-induced cardiac dysfunction by inhibiting the NLRP3 inflammasome through upregulation of the SIRT1 pathway (168). This process reduces the release of inflammatory factors and macrophage infiltration into cardiac tissues, thereby alleviating LPS-induced myocardial injury and improving cardiac function (168).

### 4.3 Phenolic acids

Resveratrol shows promise in HF treatment (169). After a 3-month treatment with resveratrol, patients with systolic HF experienced significant reductions in IL-1, IL-6, NT-proBNP, galectin-3, total cholesterol, and low-density lipoprotein cholesterol levels, along with substantial enhancements in cardiac function and quality of life (169). Resveratrol can mitigate MI/R injury (170). It mitigates cardiac inflammation, fibrosis, and apoptosis by modulating the SIRT1/p53 signaling pathway and inhibiting the NLRP3 inflammasome, thereby ameliorating MI/R-induced cardiac dysfunction (170). Furthermore, resveratrol reduced acute sympathetic stress-induced cardiac inflammation by inhibiting the Akt1/NLRP3 inflammasome pathway (171). Salvianolic acid B inhibited the activation of the NLRP3 inflammasome by regulating the SIRT1/AMPK/PGC-1α signaling pathway (172). This action leads to decreased cardiac inflammation, mitigation of mitochondrial dysfunction, and a reduction in cardiomyocyte apoptosis, ultimately exerting a cardioprotective effect in ischemic and hypoxic conditions (172). Salvianolic acid B can also ameliorate myocardial inflammation and enhance mitochondrial function by promoting mitochondrial autophagy and inhibiting the NLRP3 inflammasome, thus alleviating ISO-induced acute myocardial ischemic injury (173). Chicoric acid effectively mitigates the detrimental effects of cardiac overload (174). Specifically, it reduced ISO-induced cardiac inflammation, fibrosis, apoptosis, and mitochondrial structural damage by inhibiting the hexokinase 1(HK1)/NLRP3 inflammasome signaling pathway (174). When exposed to DOX, cardiomyocytes exhibit impaired contractile function (175). Curcumin activates the AKT/mTOR pathway, leading to a reduction in DOX-induced pyroptosis and autophagy, thereby contributing to the alleviation of cardiomyocyt\e apoptosis and cardiac dysfunction (175). Carvacrol is beneficial in attenuating LPS-induced cardiac dysfunction, and its protective effect against myocardial injury is linked to the inhibition of pyroptosis mediated by the NLRP3/caspase1/GSDMD pathway (176).

### 4.4 Quinones

After AMI, the expression of TLR4, NF-κB p65, NLRP3, IL-1β, and IL-18 increased in cardiac tissue (177). These changes lead to adverse cardiac effects such as myocardial inflammation, fibrosis, cardiomyocyte apoptosis, and cardiac dysfunction (177). Tanshinone IIA mitigated these adverse changes by inhibiting the TLR4/NF-κB p65/NLRP3 inflammasome signaling pathway, thus enhancing cardiac structure and restoring left ventricular function (177). After MI, treatment with salvianolate ameliorated interstitial fibrosis in the atria, decreased the susceptibility of the heart to atrial fibrillation, and reduced the duration of atrial fibrillation (178). Salvianolate’s cardioprotective effect was attributed to its capacity to reduce collagen deposition and attenuate inflammatory responses by inhibiting the TGF-β1/Smad2/3 and TXNIP/NLRP3 inflammasome signaling pathways (178). Emodin decreased the expression of GSDMD-NT and IL-1β by inhibiting the TLR4/MyD88/NF-κB/NLRP3 inflammasome signaling pathway, thereby mitigating myocardial inflammatory injury induced by MI/R (179). Furthermore, Emodin potentially ameliorated LPS-induced cardiac injury and dysfunction (180). This is primarily achieved by inhibiting the NLRP3 inflammasome, decreasing the levels of inflammatory cytokines, and inducing cardiomyocyte pyroptosis (180). Sodium tanshinone IIA sulfonate has demonstrated potential for the treatment of sepsis-induced myocardial dysfunction (181). In mice with LPS-induced cardiomyopathy, sodium tanshinone IIA sulfonate mitigates myocardial inflammation and enhances cardiac function by promoting autophagy and inhibiting NLRP3 inflammasome activation, leading to increased survival rates (181).

### 4.5 Phenylpropanoids

Beta-asarone reduces cardiac inflammation and diminishes the size of MI by inhibiting the NLRP3 inflammasome, thus enhancing cardiac recovery after ischemia reperfusion (182). The cardioprotective effect of cinnamaldehyde also depends on its inhibitory effects on the NLRP3 inflammasome (183). Pretreatment with Cinnamaldehyde attenuated cardiomyocyte pyroptosis and the number of TUNEL-positive cells by suppressing the expression of NLRP3, ASC, pro-caspase-1, caspase-1, and GSDMD, as well as the release of IL-18 and IL-1β, thereby alleviating MI/R injury (183). Aesculin also confers protective effects against ischemia reperfusion in the myocardium (184). It hindered the activation of the NF-κB/NLRP3 inflammasome signaling pathway by enhancing Akt and GSK3β expression, leading to reduced cardiac inflammation, enhanced mitochondrial function, reduced cardiomyocyte apoptosis, and decreased vulnerability to ventricular arrhythmias, ultimately enhancing cardiac function (184). Cinnamic acid alleviates MI/R injury by reducing the infarct size, preventing myocardial cell apoptosis, and improving cardiac diastolic function (185). The cardioprotective effects of cinnamic acid have been attributed to the suppression of NLRP3 inflammasome activation-induced pyroptosis (185).

### 4.6 Other active ingredients

In addition to the aforementioned compounds, gastrodin (186), panaxynol (187), and the ethyl acetate extract of cinnamomi ramulus (188) had been identified as having the potential to ameliorate symptoms and enhance the prognosis of HF by inhibiting the NLRP3 inflammasome. By inhibiting NLRP3/caspase-1 signaling, Gastrodin reduced the production of IL-1β (186). This mechanism alleviates the inflammatory responses in the heart and microvasculature, reduces myocardial apoptosis, and promotes capillary formation, thereby offering protection against myocardial and cardiac microvascular damage induced by MI/R (186). Panaxynol exhibits anti-inflammatory and anti-apoptotic properties (187). It demonstrated beneficial effects in mitigating MI/R injury by suppressing the HMGB1/TLR4/NF-κB/NLRP3 inflammasome signaling pathway, leading to a significant reduction in MI size and enhancement of cardiac function (187). *Cinnamomi Ramulus* also has the potential to ameliorate adverse cardiac outcomes resulting from MI/R (188). It attenuates cardiac inflammation and enhances cardiac function by reducing NLRP3 inflammasome activation and pyroptosis, thereby exerting cardioprotective effects (188).

## 5 Conclusions and prospects

The activation of the NLRP3 inflammasome is a complex process involving three distinct pathways: canonical, non-canonical, and alternative NLRP3 inflammasome activation. These activated pathways adversely affect cardiac function by promoting the progression of various pathological processes, including the exacerbation of myocardial inflammatory injury, adverse myocardial fibrosis, pathological myocardial hypertrophy, and abnormal cardiomyocyte apoptosis; inhibition of angiogenesis; and disruption of cardiac electrical signaling and energy metabolism. These factors synergistically accelerate the onset and progression of HF. In the prevention and treatment of HF, the active ingredients of TCM demonstrate significant potential. They inhibit the NLRP3 inflammasome through multiple pathways, effectively attenuating the aforementioned pathological changes and thereby improving both the structure and function of the heart. Furthermore, we found that these active ingredients are primarily concentrated in flavonoids and their glycosides, terpenes and their glycosides, phenolic acids, quinones, and phenylpropanoids. Based on these findings, we posit that there is both theoretical value and clinical significance in reviewing studies on TCM active ingredients for preventing and treating HF, with a focus on the inhibition of the NLRP3 inflammasome. This endeavor aims to lay the foundation for future research and the development of novel therapeutic agents.

Despite some progress in investigating the inhibition of the NLRP3 inflammasome by the active ingredients of TCM for the prevention and treatment of HF, various limitations and challenges persist. First, some studies lack in-depth exploration of the upstream signaling molecules that regulate the NLRP3 inflammasome. It remains unclear which specific signaling pathways the active components of TCM utilize to inhibit the NLRP3 inflammasome. Second, active ingredients of TCM may engage multiple signaling pathways to suppress the NLRP3 inflammasome, such as Astragaloside IV, Luteolin, and Resveratrol. However, there remains a shortage of comprehensive investigations into the interplay between molecular signals. Third, most current research is confined to animal and cell experiments and lacks robust clinical studies that offer evidential support, particularly high-quality randomized controlled trials. Fourth, the etiology of HF is multifaceted and includes myocardial ischemia, cardiac overload, and myocardial toxicity. Further research is necessary to verify whether there are variations in the mechanism of action and the effects of the same active ingredients in TCM on HF with different etiologies. Fifth, although the active ingredients of TCM exhibit minimal side effects and low drug resistance, HF is characterized by a protracted course of illness that may require extended dosing periods. Therefore, the safety profiles of the active ingredients in Chinese medicines require evaluation through meticulously designed clinical studies.

Considering the aforementioned limitations and challenges, future studies should conduct multidimensional validations using HF models derived from various etiological sources both *in vitro* and *in vivo*. Simultaneously, emphasis should be placed on exploring upstream signaling molecules that suppress the NLRP3 inflammasome and investigating the interactions among diverse molecular mechanisms. Furthermore, after verifying the efficacy and safety of the active ingredients in TCM in basic research, clinical trials should be conducted to assess the therapeutic potential of these components in preventing and treating HF, thereby enabling the translation of research findings into clinical applications.

## Glossary

Akt: serine/threonine protein kinase B
AMI: acute myocardial infarction
AMPK: adenosine 5’-monophosphate (AMP)-activated protein kinase
AMVMs: adult mouse ventricular myocytes
Ang II: angiotensin II
ASC: apoptosis speck-like protein containing a caspase recruitment domain
As-IV: Astragaloside IV
α-SMA: α-smooth muscle actin
ATP: adenosine triphosphate
BMDMs: bone marrow-derived macrophages
CARD: caspase activation and recruitment domain
CFs: cardiac fibroblasts
CMECs: cardiac microvascular endothelial cells
H/R: hypoxia/reoxygenation
Cx43: Connexin 43
DCM: dilated cardiomyopathy
DOX: Doxorubicin
ECM: Extracellular matrix
ECs: endothelial cells
GSDMD: gasderminD
GSDMD-NT: GSDMD N-terminal
HCMs: human cardiomyocytes
HF: heart failure
HFpEF: heart failure with preserved ejection fraction
HK1: hexokinase 1
HUVECs: human umbilical vein endothelial cells
IFN‐γ: interferon-gamma
IL: interleukin
ISO: isoproterenol
KI: knock-in
KO: knockout
K^+^: potassium ion
LADCA: left anterior descending coronary artery
LCA: left coronary artery
LPS: lipopolysaccharide
LRR: leucine rich repeat
MAPK: mitogen-activated protein kinase
MCFs: mouse cardiac fibroblasts
MCP-1: monocyte chemoattractant protein-1
MI: myocardial infarction
MI/R: myocardial ischemia/reperfusion
mtROS: mitochondrial reactive oxygen species
mtDNA: mitochondrial DNA
MyD88: myeloid differentiation primary response protein 88
NF-κB: nuclear factor-κB
NLRP3: NOD-like receptor protein 3
NMCMs: neonatal mouse cardiomyocytes
NMVMs: neonatal mouse ventricular cardiomyocytes
NRCFs: neonatal rat cardiac fibroblasts
NRCMs: neonatal rat cardiomyocytes;NRVMs, neonatal rat ventricular myocytes
OGD/R: oxygen-glucose deprivation/reoxygenation
OS: oxidative stress
pro-caspase-1: caspase-1 precursor
PYD: pyrin domain
P2X7R: P2X7 receptor
RV: right ventricular
SD: sprague dawley
SHRs: spontaneously hypertensive rats
SIRT: silent information regulator of transcription
TAC: transverse aortic constriction
TGF-β: transforming growth factor-β
TLR: toll-like receptor
TNF-α: tumor necrosis factor-α;VAs, ventricular arrhythmias
WKY: Wistar-Kyoto
WT: wild type.
