# Supplementary material for: Active ingredients of traditional Chinese medicine inhibit NOD-like receptor protein 3 inflammasome: a novel strategy for preventing and treating heart failure
Source: Front Immunol. 2025 Jan 24;16:1520482. doi: 10.3389/fimmu.2025.1520482 (PMC11802527; doi:10.3389/fimmu.2025.1520482)
Supplement: Supplementary file 1 [file Table1.docx]

**Supplementary Table S1** Database, strategy, and timing for literature search.

| Databases | Search strategy | Update time |
| --- | --- | --- |
| PubMed | ((heart failure OR myocardial remodeling OR cardiac remodeling OR ventricular remodeling OR atrial remodeling OR myocardial infarction OR myocardial ischemia reperfusion OR hypertension OR cardiomyopathy OR myocardial toxicity) AND (Chinese medicine OR herb OR herbal medicine OR active ingredient OR active compound OR natural compounds OR bioactive compound OR bioactive constituent OR active constituent OR effective constituent)) AND (NLRP3 OR Inflammasome) | December 22, 2024 |
| Web of Science | (heart failure OR myocardial remodeling OR cardiac remodeling OR ventricular remodeling OR atrial remodeling OR myocardial infarction OR myocardial ischemia reperfusion OR hypertension OR cardiomyopathy OR myocardial toxicity) And (Chinese medicine OR herb OR herbal medicine OR active ingredient OR active compound OR natural compounds OR bioactive compound OR bioactive constituent OR active constituent OR effective constituent) And (NLRP3 OR Inflammasome) | December 22, 2024 |
| Embase | ('heart failure'/exp OR 'heart failure' OR (('heart'/exp OR heart) AND ('failure'/exp OR failure)) OR 'myocardial remodeling'/exp OR 'myocardial remodeling' OR (myocardial AND remodeling) OR 'cardiac remodeling'/exp OR 'cardiac remodeling' OR (('cardiac'/exp OR cardiac) AND remodeling) OR 'ventricular remodeling'/exp OR 'ventricular remodeling' OR (ventricular AND remodeling) OR 'atrial remodeling'/exp OR 'atrial remodeling' OR (atrial AND remodeling) OR 'myocardial infarction'/exp OR 'myocardial infarction' OR (myocardial AND ('infarction'/exp OR infarction)) OR 'myocardial ischemia reperfusion' OR (myocardial AND ('ischemia'/exp OR ischemia) AND ('reperfusion'/exp OR reperfusion)) OR 'hypertension'/exp OR hypertension OR 'cardiomyopathy'/exp OR cardiomyopathy OR 'myocardial toxicity' OR (myocardial AND ('toxicity'/exp OR toxicity))) AND ('chinese medicine'/exp OR 'chinese medicine' OR (('chinese'/exp OR chinese) AND ('medicine'/exp OR medicine)) OR 'herb'/exp OR herb OR 'herbal medicine'/exp OR 'herbal medicine' OR (herbal AND ('medicine'/exp OR medicine)) OR 'active ingredient'/exp OR 'active ingredient' OR (('active'/exp OR active) AND ingredient) OR 'active compound' OR (('active'/exp OR active) AND ('compound'/exp OR compound)) OR 'natural compounds' OR (('natural'/exp OR natural) AND compounds) OR 'bioactive compound'/exp OR 'bioactive compound' OR (('bioactive'/exp OR bioactive) AND ('compound'/exp OR compound)) OR 'bioactive constituent' OR (('bioactive'/exp OR bioactive) AND constituent) OR 'active constituent' OR (('active'/exp OR active) AND constituent) OR 'effective constituent' OR (effective AND constituent)) AND (nlrp3 OR 'inflammasome'/exp OR inflammasome) | December 22, 2024 |
